# *PhOBF1*, a petunia ocs element binding factor, plays an important role in antiviral RNA silencing

**DOI:** 10.1093/jxb/erw490

**Published:** 2017-01-04

**Authors:** Daoyang Sun, Shaohua Li, Lixin Niu, Michael S Reid, Yanlong Zhang, Cai-Zhong Jiang

**Affiliations:** 1 Department of Landscape Architecture and Arts, Northwest A&F University, Yangling, Shaanxi, China; 2 Department of Plant Sciences, University of California Davis, Davis, CA, USA; 3 Crops Pathology and Genetic Research Unit, United States Department of Agriculture, Agricultural Research Service, Davis, CA, USA

**Keywords:** Phenylpropanoid pathways, salicylic acid biosynthesis, shikimate, *tobacco mosaic virus*, *tobacco rattle virus*, transcription factor, virus-induced gene silencing

## Abstract

Virus-induced gene silencing (VIGS) is a common reverse genetics strategy for characterizing the function of genes in plants. The detailed mechanism governing RNA silencing efficiency triggered by viruses is largely unclear. Here, we reveal that a petunia (*Petunia hybrida*) ocs element binding factor, *PhOBF1*, one of the basic leucine zipper (bZIP) transcription factors, was up-regulated by *Tobacco rattle virus* (TRV) infection. Simultaneous silencing of *PhOBF1* and a reporter gene, *phytoene desaturase* (*PDS*) or *chalcone synthase* (*CHS*), by TRV-based VIGS led to a failure of the development of leaf photobleaching or the white-corollas phenotype. *PhOBF1* silencing caused down-regulation of RNA silencing-related genes, including *RNA-dependent RNA polymerases* (*RDR*s), *Dicer-like RNase III enzymes* (*DCL*s), and *Argonautes* (*AGO*s). After inoculation with the TRV-*PhPDS*, *PhOBF1*-RNAi lines exhibited a substantially impaired *PDS* silencing efficiency, whereas overexpression of *PhOBF1* resulted in a recovery of the silencing phenotype (photobleaching) in systemic leaves. A compromised resistance to TRV and *Tobacco mosaic virus* was found in *PhOBF1*-RNAi lines, while *PhOBF1*-overexpressing lines displayed an enhanced resistance to their infections. Compared with wild-type plants, *PhOBF1*-silenced plants accumulated lower levels of free salicylic acid (SA), salicylic acid glucoside, and phenylalanine, contrarily to higher levels of those in plants overexpressing *PhOBF1*. Furthermore, transcripts of a number of genes associated with the shikimate and phenylpropanoid pathways were decreased or increased in *PhOBF1*-RNAi or *PhOBF1*-overexpressing lines, respectively. Taken together, the data suggest that PhOBF1 regulates TRV-induced RNA silencing efficiency through modulation of *RDR*s, *DCL*s, and *AGO*s mediated by the SA biosynthesis pathway.

## Teaser

An ocs element binding factor, *PhOBF1*, positively regulates antiviral RNA silencing mediated by salicylic acid biosynthesis and is crucial for efficient gene silencing in petunia.

## Introduction

Virus-induced gene silencing (VIGS), related to post-transcriptional gene silencing, is an attractively fast approach for degradation of homologous RNA molecules in plants (Reid *et al.*, 2009; Jiang *et al.*, 2011). This RNA silencing machinery employs a number of core components, including RNA-dependent RNA polymerases (RDRs), Dicer-like RNase III enzymes (DCLs), and Argonautes (AGOs), to implement small interfering RNA (siRNA)-mediated gene knockdown in a sequence-specific manner (Schwach *et al.*, 2005; Jaubert *et al.*, 2011). The Arabidopsis genome contains four DCLs, six RDRs, and ten AGOs (Donaire *et al.*, 2008; Zhang *et al.*, 2012), respectively contributing to the siRNA biogenesis, double-stranded RNA (dsRNA) formation, and incorporation into an RNA-induced silencing complex.

DCL1 uniquely accounts for the generation of 21-nucleotide (nt) microRNAs (miRNAs), which functions as a crucial coordinator of intricate biological processes (Bartel and Bartel, 2003). The dsRNA structures can be precisely recognized and cleaved by DCL2, DCL3, and DCL4 into 22-, 24-, and 21-nt siRNAs (Donaire *et al.*, 2008; Axtell, 2013). Depletion of DCL2, DCL3, and DCL4 enhances susceptibility to infection by RNA viruses, including *Tobacco rattle virus* (TRV) (Deleris *et al.*, 2006), *Cucumber mosaic virus* (CMV), and *Turnip crinkle virus* (TCV) (Bouché *et al.*, 2006) in Arabidopsis. It has been noted that DCL2 and DCL4 appear to impose a principle influence on virus-induced RNA silencing, whereas DCL3 acts as a minor regulator in antiviral silencing (Qu *et al.*, 2008; Garcia-Ruiz *et al.*, 2010). Plants use multiple RDR host pathways to generate viral secondary siRNAs by re-amplifying the cleaved single-stranded (ss) RNA complementary to the siRNA, which therefore favors the improvement of the silencing effect (Molnar *et al.*, 2010; Alvarado and Scholthof, 2011). Moreover, RDR1, RDR2, and RDR6 are implicated in defense against various RNA viruses via production of viral secondary siRNAs (Donaire *et al.*, 2008; Wang *et al.*, 2010). For instance, the mutants lacking RDR1, RDR2, and RDR6 exhibit a compromised resistance to *Turnip mosaic virus* (TuMV) infection in Arabidopsis (Garcia-Ruiz *et al.*, 2010). With respect to AGOs, AGO1 plays a major role in the miRNA silencing pathway (Baumberger and Baulcombe, 2005), which controls endogenous genes in terms of development, hormone regulation and stress response (Voinnet, 2009). Current evidence has uncovered the importance of AGO1 in the RNA silencing mechanism for transcriptional regulation as well as in antiviral responses (Alvarado and Scholthof, 2011). In contrast to AGO1, AGO2 seems to be a more essential factor in RNA silencing and combat for viruses. AGO2 mutation leads to an impaired resistance to invasion by CMV (Wang *et al.*, 2011), TCV (Harvey *et al.*, 2011), *Potato virus X* (PVX) (Jaubert *et al.*, 2011), and *Tomato bushy stunt virus* (Scholthof *et al.*, 2011) in Arabidopsis or *Nicotiana benthamiana.* To date, however, how these RNA silencing components are transcriptionally regulated remains largely unknown.

Salicylic acid (SA) is an endogenous phytohormone required for induction of systemic acquired resistance (Malamy *et al.*, 1990). Inoculation with viral and non-viral pathogens results in increased SA accumulation (Kumar and Klessig, 2003). Exogenous application of SA inhibits virus proliferation of CMV, *Tobacco mosaic virus* (TMV), and TCV in Arabidopsis, tomato, tobacco and hot pepper (Shang *et al.*, 2011). Previous studies revealed two separated pathways, the isochorismate and phenylalanine ammonia lyase pathways, for SA biosynthesis in Arabidopsis (D’Maris Amick Dempsey *et al.*, 2011). Both pathways initiate from chorismate, which is the ultimate product of the shikimate pathway. The aromatic amino acid phenylalanine is synthesized also through the shikimate pathway (Tzin and Galili, 2010). Some of the synthesized SAs are converted into conjugated SA glucosides (SAGs) functioning as an inactive form, from which free SA can be released (Rivas-San Vicente and Plasencia, 2011). To date, the research concerning SA has mainly focused on plant defense signaling against abiotic and biotic elicitors. Several lines of evidence support that a close correlation between SA-mediated viral defense and RNA silencing pathways may exist in plants. Exposure to SA markedly promotes the activity of NtRdRP in tobacco plants (Xie *et al.*, 2001). Silencing suppressor of CMV disrupts both RNA silencing and SA-mediated resistance to virus infection (Ji and Ding, 2001). SA-deficient transgenic tobacco plants exhibit a reduced accumulation of *Plum pox virus*-derived small RNAs, demonstrating that SA may serve as an enhancer of antiviral RNA silencing (Alamillo *et al.*, 2006). The latest findings indicate that transcripts of *RDR1*, *RDR2*, *DCL1*, and *DCL2* are up-regulated in tomato plants following *Citrus exocortis viroid* (CEVd), *Tomato mosaic virus* (ToMV) infection, and SA treatment (Campos *et al.*, 2014).

The basic leucine zipper (bZIP) proteins constitute a large transcription factor family in eukaryotes (Riechmann *et al.*, 2000). In Arabidopsis, the bZIP family is classified into ten subfamilies (A–I, S), of which the subfamily S is the largest group including 17 members (Jakoby *et al.*, 2002). AtbZIP11 belongs to subfamily S and its translation is suppressed by sucrose targeting a conserved upstream open reading frame region in the 5′ untranslated regions of mRNA (Wiese *et al.*, 2004). AtbZIP11 regulates amino acid metabolism by directly interacting with *asparagine synthetase 1* (*ASN1*) and *proline dehydrogenase 2* (*ProDH2*) (Hanson *et al.*, 2008). Activation of AtbZIP11 leads to reprogramming of amino acid and sugar metabolism, leading to increased levels of phenylalanine, tryptophan, tyrosine, sucrose, fructose, and glucose, and reduced levels of trehalose, as well as promoting expression of corresponding metabolism-associated genes (Ma *et al.*, 2011). Plant growth is inhibited in Arabidopsis plants constitutively expressing AtbZIP11 via the regulatory mechanism associating with the sucrose non-fermenting-1-related protein kinase 1 and trehalose 6-phosphate signaling systems (Ma *et al.*, 2011). In addition, AtbZIP11 specifically activates the transcription of auxin-responsive genes via recruitment of histone acetylation machinery and contributes to the modulation of auxin-mediated responses (Weiste and Dröge-Laser, 2014). Moreover, tbz17 in tobacco, lip19 and OsOBF1 in rice, and mlip15 in maize are phylogenetically close to AtbZIP11. Transcript levels of *tbz17* (Kusano *et al.*, 1998), *lip19* (Shimizu *et al.*, 2005), and *mlip15* (Kusano *et al.*, 1995) are significantly increased under low temperature stress, while *OsOBF1* shows an opposite expression pattern (Shimizu *et al.*, 2005). In *N. babacum*, transcripts of *tbz17* are up-regulated during leaf senescence (Yang *et al.*, 2001). Overexpression of *tbz17* results in enlarged leaf cells and increased sucrose levels compared with wild-type (WT) (Thalor *et al.*, 2012). But the roles of bZIP11 in antiviral RNA silencing still remain enigmatic.

In previous studies, we obtained numerous up-regulated genes, including a large number of transcription factors, during flower development through transcriptomic analysis related to flower senescence in petunia (Wang *et al.*, 2013). We have successfully used a TRV-based VIGS method to assess the function of senescence-related genes in petunia floral tissues (Chen *et al.*, 2004; Reid *et al.*, 2009; Jiang *et al.*, 2011; Chang *et al.*, 2014; Yin *et al.*, 2015). However, when the TRV-*PhCHS* report system was employed to silence several candidate genes, the inoculated plants failed to show the expected white-petal phenotype of *CHS* silencing in the purple-petal petunia plants. Of the candidate genes, we have recently reported an ethylene-responsive element binding factor, *PhERF2*, which plays a critical role in TRV-induced RNA silencing and antiviral defense via transcriptional modulation of *RDR2*, *RDR6*, *DCL2*, and *AGO2* in petunia (Sun *et al.*, 2016). In this study, we report another regulatory gene, an ocs element binding factor of bZIP transcription factors, namely *PhOBF1*. Simultaneous silencing of *PhOBF1* and reporter genes, *PDS* or *CHS*, led to a severe impairment of leaf photobleaching or white-flower phenotype in petunia. PhOBF1 appears to play a crucial role in the antiviral RNA silencing process through the regulation of the SA biosynthesis pathway.

## Materials and methods

### Plant materials and growth conditions

Petunia (*Petunia*×*hybrida*) plants were maintained in a growth chamber at 25/20 °C day/night temperatures with a 16/8 h light/dark cycle. Four-leaf-stage plantlets of cultivar ‘Primetime Blue’ were inoculated with TRV constructs to silence *PhOBF1* in leaves and floral tissues. Stable transformation was obtained from ‘Mitchell Diploid’. Four-week-old transgenic plant leaves were sampled to determine the transcript levels of *PhOBF1*, RNA silencing-related genes and genes in the SA biosynthesis pathway, as well as concentrations of SA, SAG, and phenylalanine. To further examine the impact of *PhOBF1* on silencing efficiency of the VIGS system, transgenic plants at the four-leaf stage were infiltrated with TRV-*PhPDS.* For multiple resistance analysis, 4-week-old WT and transformed seedlings were inoculated with TRV and TMV. For all studies of gene expression in virus assay, inoculated or uppermost unfolded leaves (the third or fourth leaf from terminal) were harvested and used for total RNA isolation. Tissue-specific expression analysis of *PhOBF1* was conducted using younger leaves, stems, and roots, and flowers at anthesis (separated into sepals, petals, pistils, and stamens) of 10-week-old ‘Mitchell Diploid’ plants. Corollas at day 1, 3, 5, and 7 post-anthesis were used to study *PhOBF1* expression profiles during flower senescence.

### Identification of *PhOBF1*

The *PhOBF1* cDNA fragments were isolated by RT-PCR as previously described (Chang *et al.*, 2014). Corresponding amino acids were deduced by the translate tool of ExPASy (http://web.expasy.org/translate/). Its conserved domain was identified using an NCBI web server (http://www.ncbi.nlm.nih.gov/Structure/cdd/wrpsb.cgi). Homologous proteins from petunia and other species were found through NCBI BLAST (http://blast.ncbi.nlm.nih.gov/). Multiple sequence alignment was conducted by CLUSTALW (http://www.genome.jp/tools/clustalw/). A phylogenetic tree was constructed by means of MEGA4 (version 4.0.2) software.

### Abiotic stress and hormone treatments

For the cold treatment, 3-week-old seedlings of ‘Primetime blue’ were placed in small vials with distilled water and maintained at room temperature (control) and in a 4 °C room. For the salinity and dehydration treatments, the seedlings were inserted in vials containing 100 mM NaCl and no water, respectively. For the ethylene treatment, the seedlings were treated with 10 µl l^−1^ of ethylene gas continuously in a sealed chamber. For other hormone treatments, the plants were placed in vials containing 50 μM ABA, 50 μM gibberellic acid (GA_3_), 200 μM SA, or 200 μM methyl jasmonate (MeJA). Samples were collected at 0, 3, 6, 12, and 24 h post-treatment.

### Construction of VIGS plasmids

The TRV-*PhPDS*, TRV-*PhCHS*, and TRV-*PhPDS*/*CHS* plasmids were generated previously using 138-bp *PDS* and 194-bp *CHS* fragments (Chen *et al.*, 2004). To generate the TRV-*PhPDS*/*OBF1*, TRV-*PhCHS*/*OBF1*, and TRV-*PhPDS*/*CHS*/*OBF1* constructs, the fragment of *PhOBF1* were PCR-amplified using specific primers F1R1 (Supplementary Table S1 at *JXB* online) and then cloned into three respective plasmids as mentioned above. A different fragment (fragment 2) of *PhOBF1* amplified by specific primers F2R2 (Supplementary Table S1) was cloned into TRV-*PhPDS*/*CHS*. Similarly, a *PhbZIP44* fragment was PCR-amplified to form TRV-*PhPDS*/*bZIP44*, TRV-*PhCHS*/*bZIP44*, and TRV-*PhPDS*/*CHS*/*bZIP44* constructs. Additionally, the TRV–green fluorescent protein (GFP) construct was kindly provided by Junping Gao from China Agricultural University (Beijing, China) (Tian *et al.*, 2014).

### Agro-inoculation of TRV vectors

*Agrobacterium tumefaciens* strain GV3101 was electrotransformed with TRV1 and TRV2 plasmids. The transformed bacteria were cultured in LB media with 40 mg l^−1^ kanamycin, 20 mg l^−1^ gentamicin, 10 mM MES, and 20 μM acetosyringone for 48 h at 28 °C. The cells were harvested via centrifugation and then resuspended in inoculation buffer containing 10 mM MgCl_2_, 10 mM MES, and 200 μM acetosyringone to an OD_600_ of 4.0, followed by incubation at room temperature for 3–5 h. Subsequently, the *Agrobacterium* cultures containing TRV1 and TRV2 plasmids were mixed together in a 1:1 ratio. The bacterial mixture was applied to the abaxial side of petunia leaves using a 1-ml disposal needle-free syringe (Reid *et al.*, 2009; Jiang *et al.*, 2011). The *Agrobacterium* without transformation was used as mock control.

### Semi-quantitative RT-PCR and quantitative real-time PCR

TRIzol reagent (Invitrogen, Carlsbad, CA, USA) was used to isolate total RNA from petunia leaves or flowers. Purification of RNA was performed using RNase-free DNase I (Promega, Madison, WI, USA). First-strand cDNA was synthesized from total RNA (2–5 μg) with SuperScript III reverse transcriptase (Invitrogen, Carlsbad, CA, USA). PCR was carried out using Taq DNA polymerase (Invitrogen, Carlsbad, CA, USA) according to the manufacturer’s recommendations, and resulting PCR products were analysed by electrophoresis. *26S ribosomal RNA* served as an internal control (Chen *et al.*, 2004; Reid *et al.*, 2009). Quantitative real-time PCR was performed using the SYBR Green PCR Master Mix (2X) (Applied Biosystems, Foster City, CA, USA) as previously described (Liang *et al.*, 2014). A minimum of three biological replicates were used for determining transcript abundances of genes. Statistical significance of difference was evaluated by Duncan’s multiple range test at a *P* value <0.05.

### Northern blot assay

Total RNA was extracted from the systemic petunia leaves infected with TRV vectors and mock control using TRIzol reagent (Invitrogen, Carlsbad, CA, USA). Small RNAs with low molecular mass were further isolated with a solution containing polyethylene glycol (PEG8000) and NaCl as previously described (Wang *et al.*, 2004). The ethidium bromide stained 5S rRNA was served as a loading control. Blot hybridization was conducted as previously described (Donaire *et al.*, 2008). For analysis of siRNAs derived from TRV-*PhPDS*, oligonucleotide probes corresponding to *PDS* insert (138 bp) in TRV2 vector were radiolabeled with [γ-^32^P]ATP using T4 polynucleotide kinase (Promega). Finally, relative siRNA accumulation was visualized through RNA blots exposed to autoradiographic ﬁlm.

### Generation of transgenic petunia plants

The full-length of the *PhOBF1* ORF sequence was PCR-amplified using the following primers: 5′-ATCTCGAGATGG CATCTTCTAGTGGAAA-3′ (forward) with an *Xho*I restriction site and 5′-ATGAGCTCTCAATACTGATAGAACATAT-3′ (reverse) with a *Sac*I restriction site. The fragment was cloned into pGSA1403 vector to form the overexpression construct (Chang *et al.*, 2014; Yin *et al.*, 2015). For RNAi construct, the fragment (Supplementary Fig. S1A) of *PhOBF1* cDNA was amplified using the primers F1R1 (Supplementary Table S1) with addition of a sequence harboring *Spe*I–*Asc*I and *Bam*HI–*Swa*I restriction sites in the 5′ end of forward and reverse primers, respectively. The resulting products were ligated into pGSA1285 vector in sense and antisense orientations. The plasmids for overexpression and RNAi constructs were transformed into *A. tumefaciens* strain LBA4404 by electroporation. Petunia cultivar ‘Mitchell Diploid’ was used for generation of transgenic plants using the leaf-disk transformation method (Wang *et al.*, 2013; Liang *et al.*, 2014). Positive transgenic plants were screened on MS plates supplemented with 100 mg l^−1^ kanamycin (Estrada-Melo *et al.*, 2015; Yin *et al.*, 2015).

### Measurement of SA, SAG, and phenylalanine

Petunia leaves (0.2–0.5 g) were ground to fine powder in liquid nitrogen using a mortar and pestle. Determination of free SA and SAG levels was performed using a method previously described (Lee *et al.*, 2011) with some minor changes. Methanol–formic acid (1 ml; 95:5) was used to extract the leaf tissues. Samples were vortexed and sonicated for 20 min for a complete homogenization. The mixture was centrifuged at 15 000 *g* for 10 min at 4 °C. The supernatant was filtered through a 0.45 μm filter and then freeze-dried in a vacuum. The pellet was resuspended in 1 ml of 5% trichloroacetic acid. Free SA was extracted organically by supplementing 1 ml of ethylacetate–cyclopentane–isopropanol (50:50:1). The upper phase containing free SA was collected and analysed by the high-performance liquid chromatography (HPLC). The SAG-containing aqueous phase after free SA extraction was exposed to acidification treatment by hydrochloric acid to pH 1.0. Subsequent boiling of acidified solution for 30 min was implemented for the release of SA from conjugated forms. The released SA was measured as described above. For quantification of phenylalanine, the ground powder of leaves was extracted with 1 ml 60% methanol (v/v), 0.1% phosphoric acid (v/v) with 2% 3-methylsalicylic acid (w/v). Following sonication, centrifugation, and filtration, the clear solution was injected into a C_18_ column for HPLC separation as reported previously (Janzik *et al.*, 2005). The HPLC assay was performed using an Agilent chromatograph (model 1100, Agilent Technologies, Santa Clara, CA, USA), equipped with a fluorescence detector. The pure SA and phenylalanine (Sigma-Aldrich, St Louis, MO, USA) were dissolved and used as reference standards.

### Inoculation with TMV

Infectious sap in 0.1 M phosphate buffer (pH 7.0, 1:4 w/v) was prepared from TMV-infected leaves of *N. benthamiana.* Prior to inoculation, the fully expanded leaves of 4-week-old healthy petunia plantlets were dusted with 400 mesh carborundum powder. The TMV sap was used to inoculate the leaves by a mechanical method (Hull, 2009). Relative virus accumulation was checked by quantitative real-time PCR analysis of transcript levels of TMV*-CP*, encoding coat protein of TMV.

## Results

### TRV infection, abiotic stresses and hormone treatments induce *PhOBF1* expression

**Fig. 1.**
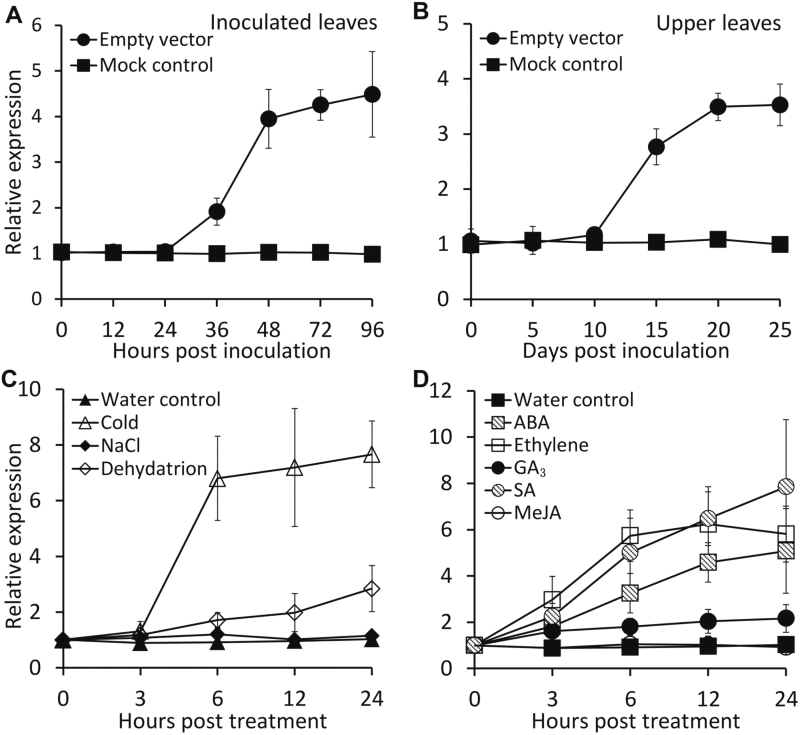
Induction of *PhOBF1* expression by TRV infection, abiotic stresses and hormone treatments in petunia leaves. (A, B) Quantitative real-time PCR analysis of *PhOBF1* transcript levels in inoculated (A) and systemically infected upper (B) leaves with TRV at different time points. Four-week-old WT plants were inoculated with *Agrobacterium* bearing no TRV vector (mock control) or a TRV empty vector. (C, D) Quantitative real-time PCR analysis of *PhOBF1* transcript levels in response to abiotic stresses (C) and hormones (D) at different time points. Three-week-old petunia seedlings were placed in vials with water at room temperature (control) or in a 4 °C room (cold), or without water at room temperature (dehydration), or with 100 mM NaCl, 50 μM ABA, 50 μM GA_3_, 200 μM SA, or 200 μM MeJA, or exposed to 10 μl l^−1^ ethylene treatment. Transcript abundances were standardized to *26S rRNA*. Error bars represent standard error of the mean from three biological replicates.

A 1029-bp cDNA corresponding to a putative ocs element binding factor 1 (*OBF1*) gene was isolated from a library constructed from petunia flower (Wang *et al.*, 2013). The deduced protein, annotated as PhOBF1, was phylogenetically closed to tomato (*Solanum lycopersicum*) SlOBF1, potato (*S. tuberosum*) StOBF1, and Arabidopsis AtbZIP11. These proteins share a conserved basic leucine zipper domain with a length of 63 amino acids (Supplementary Fig. S1). *PhOBF1* was constitutively expressed in leaves and roots at a higher level than in stem and floral tissues (Supplementary Fig. S2). To study the impact of TRV infection on *PhOBF1* expression, the *Agrobacterium* bearing TRV empty vector was used to inoculate the leaves of four-leaf-stage WT petunia seedlings. Transcript abundances of *PhOBF1* increased remarkably at 36 h post-inoculation (hpi) in inoculated leaves and 15 d post-inoculation (dpi) in systemically infected upper leaves, respectively (Fig. 1A, B). By contrast, the inoculation with non-transformed *Agrobacterium* (mock control) did not alter *PhOBF1* expression levels (Fig. 1A, B). Furthermore, transcript levels of *PhOBF1* increased following exposure to low temperature and drought stresses (Fig. 1C) and was also up-regulated following ABA, ethylene, GA_3_, and SA treatments (Fig. 1D).

### Simultaneous silencing of *PhOBF1* and reporter genes disrupts VIGS efficiency

**Fig. 2.**
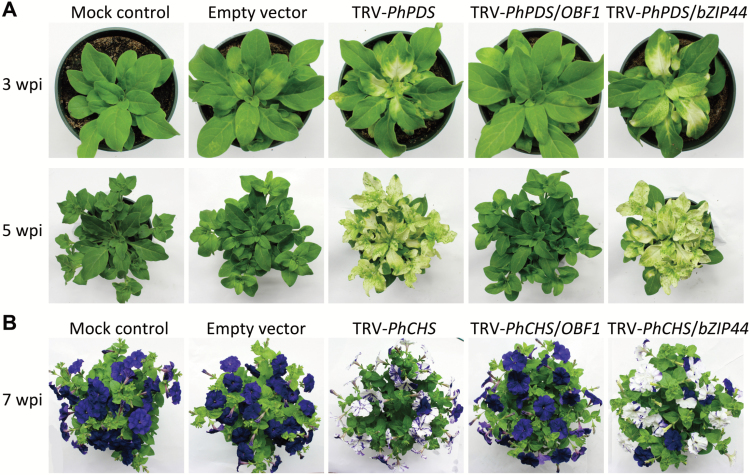
Failed development of *PDS*-silenced leaf photobleaching and *CHS*-silenced white-corollas phenotypes in plants with *PhOBF1*-VIGS silencing. Representative phenotypes of WT plants at 3, 5 (A), and 7 (B) weeks post-inoculation (wpi) with *Agrobacterium* bearing no TRV vector (mock control), or a TRV empty vector, TRV-*PhPDS* and TRV-*PhCHS*, TRV-*PhPDS*/*OBF1* and TRV-*PhCHS*/*OBF1*, TRV-*PhPDS*/*bZIP44* and TRV-*PhCHS*/*bZIP44* constructs, respectively.

To investigate the function of *PhOBF1*, TRV-based VIGS with visual reporters of *PDS* and *CHS* was employed to knockdown *PhOBF1* expression in the leaves and flowers of petunia, respectively. The plants inoculated with mock and empty vector displayed the normal WT phenotype with green leaves (Fig. 2A) and purple corollas (Fig. 2B). TRV-*PhPDS*-infected systemic leaves showed a typical *PDS*-silenced photobleaching phenotype. However, TRV-*PhPDS*/*OBF1*-infected systemic leaves failed to show a photobleaching phenotype at 3 and 5 weeks post-inoculation (wpi) (Fig. 2A). To examine whether the failed photobleaching phenotype is only associated with the *PhOBF1* gene, a cDNA fragment from a paralog of *PhOBF1*, *PhbZIP44* (Supplementary Fig. S1B) was used for comparison. The plants infected with TRV-*PhPDS*/*bZIP44* showed a *PDS*-silenced photobleaching phenotype (Fig. 2A). To further confirm this finding, another visual reporter gene, *CHS*, was introduced. The *CHS*-silenced white-corollas phenotype, as shown in TRV-*PhCHS* and TRV-*PhCHS*/*bZIP44*, was not observed in the flowers infected by TRV-*PhCHS*/*OBF1* at 7 wpi (Fig. 2B). Furthermore, the 3′ end fragment (Supplementary Fig. S1A) of *PhOBF1* cDNA was inserted into a tandem construct TRV-*PhPDS*/*CHS* for VIGS assay. Both systemic leaves and flowers from TRV-*PhPDS*/*CHS*/*OBF1* (F2)-infected plants did not show *PDS*-photobleaching and *CHS*-white silencing phenotypes during the entire growth periods (Supplementary Fig. S3).

### VIGS silencing of *PhOBF1* does not suppress virus proliferation

**Fig. 3.**
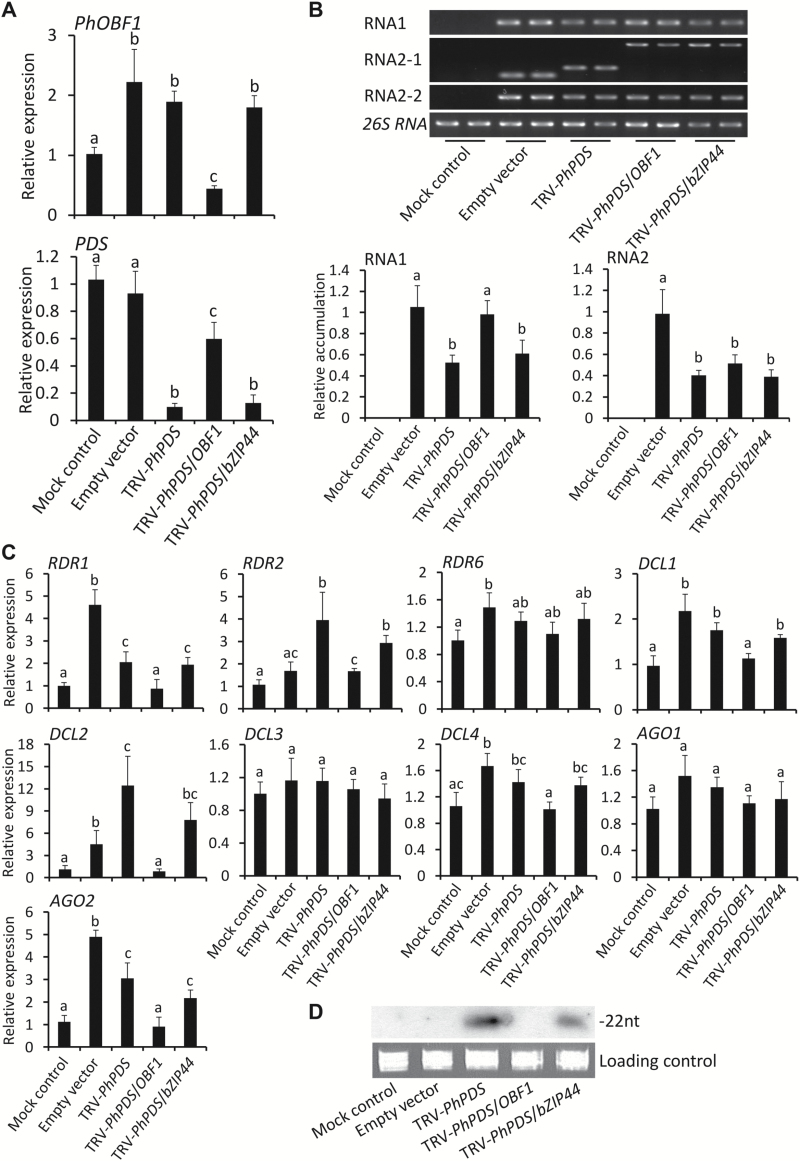
Expression of *PhOBF1*, *PDS*, and RNA silencing-related genes and accumulation of TRV and siRNAs in the leaves of agro-infiltrated plants. (A) Quantitative real-time PCR analysis of *PhOBF1* and *PDS* transcript levels in uppermost leaves of plants at 3 weeks post-inoculation (wpi) with *Agrobacterium* bearing a TRV empty vector, TRV-*PhPDS*, TRV-*PhPDS*/*OBF1*, and TRV-*PhPDS*/*bZIP44* constructs. Non-transformed *Agrobacterium* was used as mock control. Transcript abundances were standardized to *26S rRNA*. (B) Semi-quantitative RT-PCR and quantitative real-time PCR analysis of TRV RNA1 and RNA2 (RNA2-1, -2) accumulation levels in uppermost leaves of plants at 3 wpi. Primers for RNA2-1 were designed based on the region outside of multiple cloning sites (MCS) in the vector and resulted product sizes were dependent on the inserted fragments of *PDS*, *PhOBF1*/*PDS*, and *PhOBF1*/*bZIP44*, while RNA2-2 targets the region upstream of MCS and produced the same sizes of products. (C) Quantitative real-time PCR analysis of transcript levels of RNA silencing-related genes, including *RDR1*, *RDR2*, *RDR6*, *DCL1*, *DCL2*, *DCL3*, *DCL4*, *AGO1*, and *AGO2*, in uppermost leaves of plants at 3 wpi. *26S rRNA* was used as internal control. (D) Northern-blot analysis of TRV*-PhPDS*-derived siRNA levels in uppermost leaves of plants at 3 wpi. ^32^P-labeled oligonucleotide probes correspond to *PDS* insert sequence. Ethidium bromide-stained *5S rRNA* is indicated as a loading control. Error bars represent standard error of the mean from three biological replicates. Different letters indicate statistical significance as calculated by Duncan’s multiple range test at *P* < 0.05.

To understand why the inclusion of the *PhOBF1* fragment in TRV-*PhPDS* and TRV-*PhCHS* constructs severely impaired the *PDS*-silenced leaf photobleaching and *CHS*-silenced white-corollas phenotypes, we analysed transcript abundances of *PhOBF1* and *PDS*, and accumulation levels of TRV RNA1 and RNA2 in uppermost leaves of plants at 3 wpi. *PhOBF1* transcripts were up-regulated in the leaves from plants infected with TRV constructs except TRV-*PhPDS*/*OBF1* (Fig. 3A). The *PhOBF1* expression levels in leaves infected with TRV-*PhPDS*/*OBF1* were significantly lower than that in mock-treated leaves and other TRV construct-infected leaves (Fig. 3A), suggesting the partial knockdown of *PhOBF1* expression. When examining *PDS* transcripts, a significantly marked reduction (greater than 90%) was found in photobleached leaves from plants infected with TRV-*PhPDS* and TRV-*PhPDS*/*bZIP44* (Fig. 3A). However, only about a 40% reduction in *PDS* abundance was observed in leaves from TRV-*PhPDS*/*OBF1*-infected plants, compared with mock and empty vector controls (Fig. 3A).

Semi-quantitative RT-PCR analysis revealed that when two primer pairs covering multiple cloning sites (MCS) (RNA2-1) and a region upstream of MCS (RNA2-2) were used (Supplementary Table S1), DNA fragments carrying *PhOBF1* and *PDS* inserts were detected in TRV-*PhPDS*/*OBF1*-infected leaves (Fig. 3B), suggesting that the *PhOBF1* silencing did not suppress the TRV replication or movement. Quantitative real-time analysis indicated that the accumulation of TRV RNA1 in empty vector- and TRV-*PhPDS*/*OBF1*-infected leaves was significantly higher than that in the leaves of plants agro-infiltrated with TRV-*PhPDS* and TRV-*PhPDS*/*bZIP44* constructs (Fig. 3B).

### VIGS silencing of *PhOBF1* inhibits expression of RNA silencing-related genes and accumulation of siRNAs

To further understand failed detection of *PDS* and *CHS* silencing phenotypes in TRV-*PhPDS*/*OBF1*- and TRV-*PhCHS*/*OBF1*-infected plants, we examined transcript abundances of genes associated with the RNA silencing pathway. RDRs, DCLs and AGOs are indispensable components participating in dsRNA generation, cleavage and siRNA binding in the RNA silencing pathway (Burch-Smith *et al.*, 2006; Gould and Kramer, 2007; Jaubert *et al.*, 2011; Tian *et al.*, 2014). We measured the transcript abundances of *RDR*s (*1*, *2*, *6*), *DCL*s (*1*–*4*) and *AGO*s (*1*, *2*) in uppermost leaves of plants at 3 wpi. The majority of the selected genes, except *RDR6*, *DCL3*, and *AGO1*, were significantly up-regulated in the leaves from empty vector-, TRV-*PhPDS*- or TRV-*PhPDS*/*bZIP44*-infected plants but not in TRV-*PhPDS*/*OBF1*-infected plants (Fig. 3C).

To determine TRV-*PhPDS*-derived siRNA abundance, northern blot analysis was performed using a DNA fragment of the *PDS* reporter as probe. No signals were detectable in the leaves from TRV-*PhPDS*/*OBF1*-infected plants. By comparison, one exclusive band with a length of 22 nt was detected in the leaves from plants infected with TRV-*PhPDS* and TRV-*PhPDS*/*bZIP44* (Fig. 3D).

### *PhOBF1* silencing by RNAi impairs VIGS efficiency

**Fig. 4.**
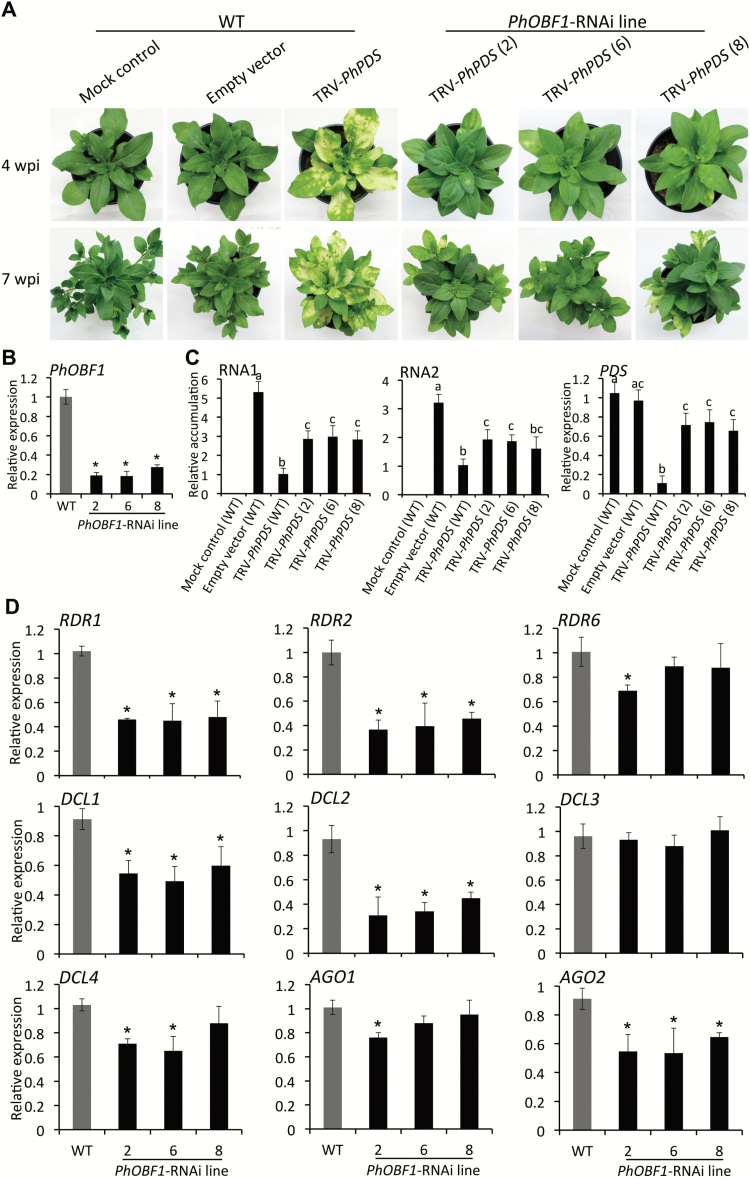
Impairment of leaf photobleaching phenotype of *PDS* silencing in *PhERF2*-RNAi lines inoculated with *Agrobacterium* bearing TRV-*PhPDS*. (A) Representative phenotypes of WT and *PhOBF1*-RNAi lines (2, 6 and 8) inoculated with non-transformed *Agrobacterium* (mock control), or *Agrobacterium* bearing a TRV empty vector and TRV-*PhPDS* construct. Photographs were taken at 4 and 7 weeks post-inoculation (wpi). (B) Quantitative real-time PCR analysis of transcript abundances of *PhOBF1* in uppermost leaves of 4-week-old WT and *PhOBF1*-RNAi lines (2, 6 and 8). Expression levels were normalized to *26S rRNA*. (C) Quantitative real-time PCR analysis of TRV (RNA1 and RNA2) accumulation and *PDS* transcript levels in uppermost leaves of WT and *PhOBF1*-RNAi lines (2, 6 and 8) at 4 wpi. Accumulation and transcript levels were standardized to *26S rRNA*. (D) Quantitative real-time PCR analysis of transcript abundances of RNA silencing-related genes, including *RDR1*, *RDR2*, *RDR6*, *DCL1*, *DCL2*, *DCL3*, *DCL4*, *AGO1*, and *AGO2*, in uppermost leaves of 4-week-old WT and *PhOBF1*-RNAi lines (2, 6 and 8). *26S rRNA* was used as internal standard. Error bars represent standard error of the mean from three biological replicates. Significance of difference was calculated using Duncan’s multiple range test (*P*<0.05) and shown as asterisks or different letters.

To better dissect PhOBF1’s function, transgenic petunia plants with down-regulated *PhOBF1* expression by RNAi were generated (lines 2, 6 and 8) and infected with TRV constructs. A striking impairment of the photobleaching phenotype was detected in the leaves of the transgenic plants infected with TRV-*PhPDS* reporter constructs (Fig. 4A), confirming the failed photobleaching phenotype in the VIGS assay. Transcript levels of *PhOBF1* in *PhOBF1*-RNAi lines were reduced by 70–80%, compared with WT (Fig. 4B). Accumulation levels of TRV RNA1 and RNA2 were higher in *PhOBF1*-silenced lines than that in WT plants infected with TRV-*PhPDS* (Fig. 4C). Following infection with TRV-*PhPDS*, approximately 90% reduction in *PDS* transcripts was found in WT leaves, whereas only 20–30% reduction in *PDS* transcripts was detected in *PhOBF1*-RNAi lines (Fig. 4C). Silencing of *PhOBF1* led to significant down-regulation of *RDR1*, *RDR2*, *DCL1*, *DCL2*, and *AGO2* in *PhOBF1*-RNAi, compared with the control lines (Fig. 4D). Transcript levels of *RDR6*, *DCL4*, and *AGO1* were slightly reduced in certain *PhOBF1*-RNAi lines (Fig. 4D). *PhOBF1* silencing did not affect transcript abundances of *DCL3* (Fig. 4D).

### *PhOBF1* overexpression restores silencing phenotype

**Fig. 5.**
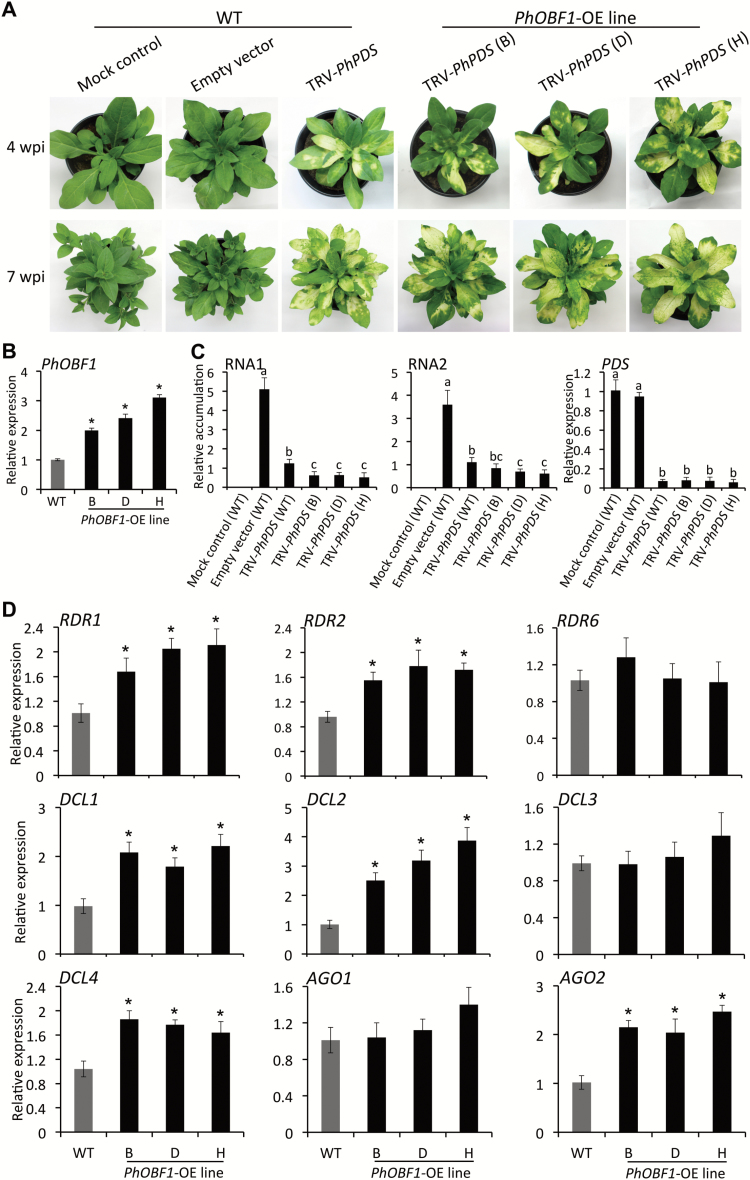
Leaf photobleaching phenotype of *PDS* silencing in *PhOBF1*-overexpressing lines inoculated with *Agrobacterium* bearing TRV-*PhPDS*. (A) Representative phenotypes of WT and *PhOBF1*-overexpressing (OE) lines (B, D and H) inoculated with *Agrobacterium* bearing no TRV vector (mock control), or a TRV empty vector and TRV-*PhPDS* construct. Photographs were taken at 4 and 7 weeks post-inoculation (wpi). (B) Quantitative real-time PCR analysis of expression levels of *PhOBF1* in the leaves of WT and *PhOBF1*-OE lines (B, D and H). Samples were harvested from uppermost leaves of 4-week-old plants. *26S rRNA* was used as normalization control. (C) Quantitative real-time PCR analysis of TRV (RNA1 and RNA2) accumulation and *PDS* transcript abundances in uppermost leaves of WT and *PhOBF1*-OE lines (B, D and H) 4 wpi. *26S rRNA* was used as internal control. (D) Quantitative real-time PCR analysis of transcript abundances of RNA silencing-related genes, including *RDR1*, *RDR2*, *RDR6*, *DCL1*, *DCL2*, *DCL3*, *DCL4*, *AGO1*, and *AGO2*, in uppermost leaves of 4-week-old WT and *PhOBF1*-OE lines (B, D and H). Transcript levels were standardized to *26S rRNA*. Error bars represent standard error of the mean from three biological replicates. Asterisks or different letters indicate statistical significance using Duncan’s multiple range test at *P*<0.05.

To further study the function of PhOBF1 in the RNA silencing process, we generated *PhOBF1*-overexpressing (OE) lines (B, D and H) in petunia. A prominent *PDS*-silenced leaf photobleaching phenotype emerged at 4 and 7 wpi in systemic leaves of WT and OE lines infected with TRV-*PhPDS* (Fig. 5A). A two- to three-fold increase in *PhOBF1* transcript levels was detected in three independent lines, compared with WT (Fig. 5B). TRV RNA1 and RNA2 accumulation was significantly decreased in *PhOBF1*-OE lines compared with WT (Fig. 5C). A dramatic and significant decrease in *PDS* transcript levels was found in the uppermost photobleached leaves of control and *PhOBF1*-OE lines infected with TRV-*PhPDS* at 4 wpi (Fig. 5C). Moreover, overexpression of *PhOBF1* significantly increased the transcript levels of *RDR1*, *RDR2*, *DCL1*, *DCL2*, *DCL4*, and *AGO2* in all transgenic lines (Fig. 5D). Overexpression of *PhOBF1* did not affect transcript abundances of *RDR6*, *DCL3*, and *AGO1.*

### *PhOBF1* affects plant defense against TRV and TMV

**Fig. 6.**
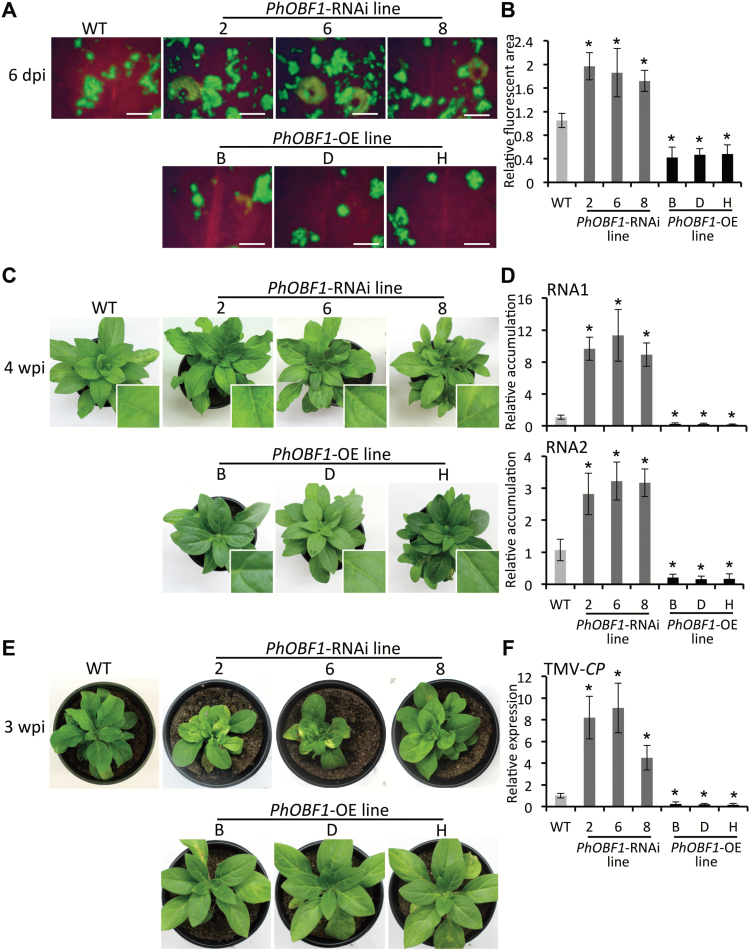
Contribution of PhOBF1 to petunia plant defense against TRV and TMV. GFP fluorescent foci (A) and relative fluorescent area (B) in inoculated leaves of WT, *PhOBF1*-RNAi (2, 6 and 8), and *PhOBF1*-overexpressing (OE) (B, D and H) lines at 6 days post-inoculation (dpi) with *Agrobacterium* bearing a TRV-GFP construct. Photographs were taken under UV light. Scale bars (A): 5 mm. Images of fluorescent area from three plants were analysed using Photoshop software. (C) Disease symptoms of WT, *PhOBF1*-RNAi (2, 6 and 8) and *PhOBF1*-OE (B, D and H) lines at 4 weeks post-inoculation (wpi) with *Agrobacterium* bearing a TRV empty vector. The insets are magnified views of viral symptoms in uppermost leaves infected with TRV. (D) Quantitative real-time PCR analysis of TRV (RNA1 and RNA2) accumulation levels in systemically infected (uppermost) leaves of WT, *PhOBF1*-RNAi (2, 6 and 8), and *PhOBF1*-OE (B, D and H) lines at 4 wpi. Transcript abundances were normalized to *26S rRNA*. (E) Disease symptoms of WT, *PhOBF1*-RNAi (2, 6 and 8), and *PhOBF1*-OE (B, D and H) lines at 3 wpi with TMV. (F) Quantitative real-time PCR analysis of transcript levels of TMV-*CP*, encoding TMV coat protein, in systemically infected (uppermost) leaves of WT, *PhOBF1*-RNAi (2, 6 and 8), and *PhOBF1*-OE (B, D and H) lines at 3 wpi. *26S rRNA* was used as an internal control. Error bars represent standard error of the mean from three biological replicates. Asterisks denote statistical significance as determined by Duncan’s multiple range test at *P*<0.05.

To examine the role of PhOBF1 in plant defense against viral infections, a TRV vector expressing green fluorescent protein (TRV-GFP) (Tian *et al.*, 2014) was used to inoculate transgenic and WT leaves to visualize the accumulation of TRV under UV irradiation. At 6 dpi, the fluorescent area was visibly larger in *PhOBF1*-RNAi but smaller in *PhOBF1*-OE lines than in the control line (Fig. 6A, B). Furthermore, TRV empty vector was used for inoculation to observe virus symptom development in the transgenic and WT plants. Compared with WT, *PhOBF1*-RNAi lines exhibited a severer leaf mottling, chlorosis and curling symptoms at 4 wpi with TRV, whereas the virus symptoms were considerably reduced in *PhOBF1*-OE lines (Fig. 6C). Consistent with that, higher accumulation levels of TRV RNA1 and RNA2 were found in *PhOBF1*-RNAi lines. On the other hand, lower accumulation levels were detected in the plants overexpressing *PhOBF1* than in WT plants (Fig. 6D and Supplementary Table S2). Furthermore, the WT plants inoculated with TMV developed a symptom shown as slightly mottled and curled leaves, while leaf yellowing, necrosis, and dwarfing were observed in *PhOBF1*-RNAi lines. A much milder virus symptom was observed in *PhOBF1*-OE lines than in WT plants (Fig. 6E). At 3 wpi, *PhOBF1*-RNAi lines accumulated more transcripts of TMV*-CP*, encoding coat protein of TMV, but fewer TMV*-CP* transcripts were detected in *PhOBF1*-OE lines than in the control line (Fig. 6F and Supplementary Table S2).

### *PhOBF1* modulates the biosynthesis of SA

**Fig. 7.**
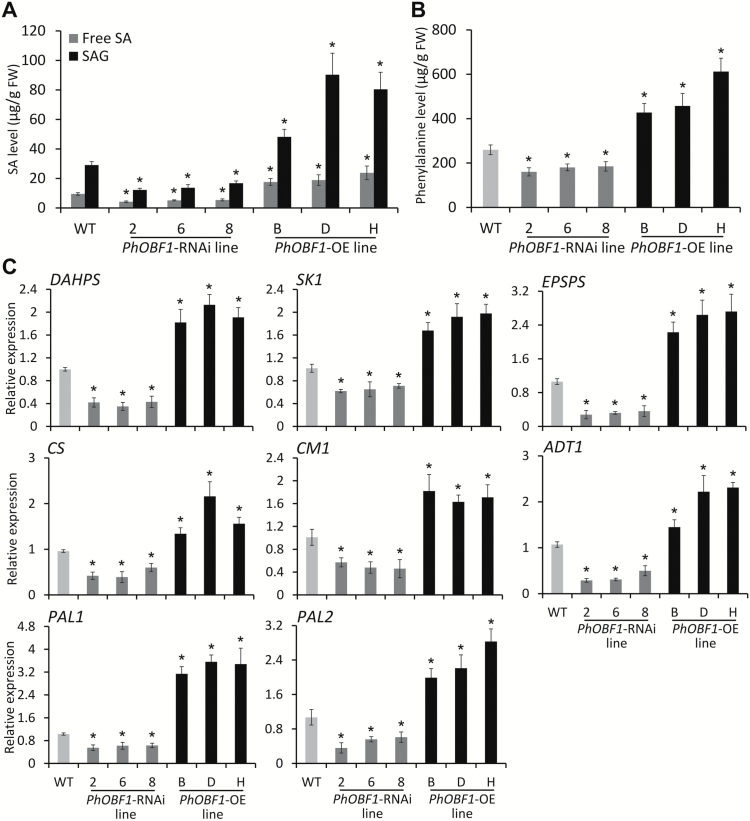
Involvement of PhOBF1 in the biosynthesis of salicylic acid. (A, B) Free SA, SA glucoside (SAG), and phenylalanine levels in the leaves of WT, *PhOBF1*-RNAi (2, 6 and 8), and *PhOBF1*-overexpressing (OE) (B, D and H) lines. Samples were harvested from all the leaves of 4-week-old WT and transgenic seedlings. (C) Quantitative real-time PCR analysis of transcript abundances of genes, including *DAHPS*, *SK1*, *EPSPS*, *CS*, *CM1*, *ADT1*, *PAL1*, and *PAL2*, in the shikimate and phenylpropanoid pathways in the leaves of WT, *PhOBF1*-RNAi (2, 6 and 8), and *PhOBF1*-OE (B, D and H) lines. Samples were harvested from the uppermost leaves of 4-week-old WT and transgenic seedlings. Expression levels were normalized to *26S rRNA*. Error bars represent standard error of the mean from three biological replicates. Asterisks indicate statistical significance using Duncan’s multiple range test at *P*<0.05.

Since the latest evidence suggests that transcripts of *RDR1*, *RDR2*, *DCL1*, and *DCL2* are up-regulated in tomato plants following CEVd, ToMV infection, and SA treatment (Campos *et al.*, 2014), we decided to examine the expression profile of RNA silencing-related genes in petunia plants treated with exogenous SA, and to measure the free SA, SAG, and phenylalanine contents in WT and transgenic plants. SA treatment resulted in a dramatic induction of *RDR1*, *RDR2*, *DCL1*, *DCL2*, *DCL4*, and *AGO2* transcripts in petunia (Supplementary Fig. S4). Importantly, concentrations of free SA and conjugated SA were significantly reduced in *PhOBF1*-RNAi lines, but increased in *PhOBF1*-OE lines, compared with WT (Fig. 7A). Transgenic lines also showed similar alteration in concentrations of phenylalanine (Fig. 7B). Furthermore, we determined transcript abundances of the core genes in the shikimate and phenylpropanoid pathways that are associated with these biochemical changes. We found that transcript abundances of genes such as *3-deoxy-d-arabino-heptulosonate-7-phosphate synthase* (*DAHPS*), *shikimate kinase* (*SK1*), *5-enolpyruvylshikimate 3-phosphate synthase* (*EPSPS*), *chorismate synthase* (*CS*), *chorismate mutase 1* (*CM1*), *arogenate dehydratase 1* (*ADT1*), *PAL1*, and *PAL2* were significantly down-regulated in *PhOBF1*-RNAi lines, but up-regulated in *PhOBF1*-OE lines (Fig. 7C). These results suggested that PhOBF1 may play important roles in the regulation of the RNA silencing pathway through biosynthesis of the hormone SA.

## Discussion

**Fig. 8.**
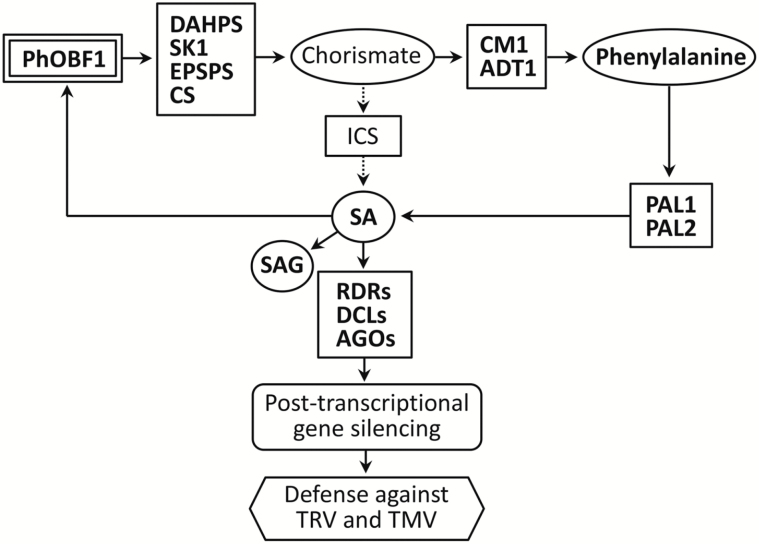
A proposed model for PhOBF1 function in antiviral RNA silencing. PhOBF1 positively regulates the genes in the shikimate and phenylpropanoid pathways, as well as phenylalanine and SA accumulation. Ultimately, PhOBF1 contributes to extensive resistance to TRV and TMV through SA-mediated post-transcriptional gene silencing. Solid arrows indicate well-described relationships, and dotted arrows indicate undescribed steps in this work. The SA, SAG, phenylalanine, and related genes analysed in *PhOBF1*-RNAi and -OE lines are shown in bold.

In this study, our data suggest that the ocs element binding factor PhOBF1 positively affects TRV-induced RNA silencing efficiency via the SA biosynthesis pathway (Fig. 8). OBF proteins bind to a class of DNA promoter sequences known as ocs elements or ACGT elements, which are essential for expression of bacterial and viral pathogen genes (Katagiri and Chua, 1992), and genes involved in the plant defense response (Ellis *et al.*, 1993; Chen and Singh, 1999). OBFs belong to a specific group of the bZIP transcription factor family in plants. A total of 75 members of the bZIP family have been identified in Arabidopsis (Jakoby *et al.*, 2002). The bZIP transcription factors induce the expression of genes whose promoter regions commonly share a core ACGT element, such as the G-box motif (CACGTG) (Hanson *et al.*, 2008). They participate in a variety of plant-specific processes, including pathogen resistance, stress response, light signaling, seed maturation and germination, and flower development (Jakoby *et al.*, 2002; Nijhawan *et al.*, 2008).

We have been investigating the role of regulatory genes, differentially expressed during petunia flower development, in the control of the corolla senescence process (Wang *et al.*, 2013). We employed the TRV-based VIGS method to silence these candidate genes, including *bZIP*s, to functionally study flower senescence-associated genes (Chang *et al.*, 2014; Yin *et al.*, 2015). When inoculated with TRV construct carrying *PhOBF1* and reporter gene (*PDS* or *CHS*) fragments, the purple-flower petunia cultivar plants were incapable of developing the *PDS*- or *CHS*-silenced white leaves or flowers throughout the entire growth period (Fig. 2A, B), suggesting a dramatic reduction in VIGS efficiency. *PhOBF1* transcripts were partially down-regulated in the leaves from TRV-*PhPDS*/*OBF1*-infected plants, in which a reduction by approximately 40% in *PDS* levels occurred, compared with up to 90% reduction in *PDS* levels in photobleached leaves in the control (Fig. 3A). These data led us to hypothesize that PhOBF1 probably functions as an important transcriptional regulator of antiviral RNA silencing.

RNA silencing triggered by virus dsRNA requires a few key components such as RDRs, DCLs and AGOs in plants (Costa *et al.*, 2013; Kasai *et al.*, 2013). Our data showed that the abundances of some RNA silencing-related genes, including *RDR1*, *RDR2*, *DCL1*, *DCL2*, *DCL4*, and *AGO2*, were decreased in the *PhOBF1*-silenced plants (Figs 3C and 4D) or increased in the *PhOBF1*-overexpressing transgenic plants (Fig. 5D). It is quite likely that PhOBF1 contributes to the RNA silencing response by modulating the transcription of these genes.

RNA silencing serves as one type of antiviral mechanism, and exerts virus-derived siRNAs (vsiRNAs) as guides to target endogenous mRNA and simultaneously viral RNA (Ma *et al.*, 2015). Interference with the RNA silencing mechanism causes an impairment of resistance to diverse viruses. Many studies have been performed to examine the impact of key genes in the silencing complex on virus resistance. For example, mutation in the *DCL* genes results in more susceptibility to TCV and CMV (Bouché *et al.*, 2006), TRV (Donaire *et al.*, 2008), TMV (Lewsey and Carr, 2009), TuMV (Garcia-Ruiz *et al.*, 2010), and PVX (Andika *et al.*, 2015). A novel argonaute protein, AGO18, has been identified as conferring a broad-spectrum virus resistance in rice (Wu *et al.*, 2015). Given PhOBF1’s importance in RNA silencing, it is expected that PhOBF1 is highly likely to be involved in plant resistance against a wide range of viral pathogens. Our findings of the compromised and enhanced resistance to TRV and TMV infections in *PhOBF1*-silenced and -overexpressing transgenic lines of petunia, respectively (Fig. 6), support this notion, and further prove the hypothesis that PhOBF1 plays a pivotal role in the antiviral RNA silencing process. Moreover, we observed quite strong effects on VIGS silencing of reporter genes (*PDS* or *CHS*) and antiviral responses given the moderate down-regulation or overexpression of *PhOBF1*. The altered transcripts of multiple RNA silencing-related genes by *PhOBF1* probably contribute to these strong effects. *DCL*s and *AGO*s play different roles in antiviral RNA silencing, and the mutant lines *ago1-27* and *dcl2*/*dcl3*/*dcl4* infected with TRV-*PhPDS* show impaired leaf photobleaching and increased TRV accumulation (Ma *et al.*, 2015). But it appears that those single or triple mutants have a relatively mild impact on gene silencing and antiviral defense compared with the *PhOBF1* silencing or overexpression. As a powerful transcription factor, PhOBF1 may greatly affect the antiviral RNA silencing process by regulating other critical factors, such as SA.

Many pieces of evidence have revealed a close association between antiviral RNA silencing and the plant endogenous hormone SA. Both ToMV and CEVd infections promote the accumulation of SA (Bellés *et al.*, 1999), as well as transcript levels of a number of *RDR* and *DCL* genes (Campos *et al.*, 2014). Exogenous SA treatment reduces the infection caused by *Alfalfa mosaic virus* (AlMV) (Van Huijsduijnen *et al.*, 1986), TMV (Chivasa *et al.*, 1997; Murphy and Carr, 2002; Shang *et al.*, 2011), CMV and TCV (Shang *et al.*, 2011), and PVX (Falcioni *et al.*, 2014). Degradation of SA in tobacco results in a reduced accumulation of virus-derived siRNAs and enhanced virus multiplication (Alamillo *et al.*, 2006). Moreover, exogenous SA treatment not only increases the levels of *RDR1* in Arabidopsis or tobacco (Yang *et al.*, 2004; Quilis *et al.*, 2008), but also *RDR2*, *DCL1*, and *DCL2* transcript abundances in tomato (Campos *et al.*, 2014). Our observations of significantly increased expression levels of *RDR1*, *RDR2*, *DCL1*, *DCL2*, *DCL4*, and *AGO2* in petunia following treatment with SA (Supplementary Fig. S4) are consistent with these findings. In addition, we found that the impaired and recovered *PDS*-silenced leaf photobleaching phenotypes in *PhOBF1*-silenced and -overexpressing petunias were accompanied by reduced and increased endogenous SA levels, respectively (Fig. 7A). It seems possible that SA serves as an intermediate signal linking PhOBF1 with the antiviral RNA silencing pathway. Future studies will determine if exogenous SA treatment can recover the impaired *PDS*-silenced leaf photobleaching or *CHS*-silenced white-corollas phenotype in *PhOBF1*-silenced petunia plants inoculated with TRV constructs carrying reporter genes (*PDS* or *CHS*).

SA biosynthesis involves the shikimate and phenylpropanoid pathways, which are controlled by some critical genes, such as *DAHPS*, *SK1*, *EPSPS*, *CS*, *CM1*, *ADT1*, *PAL1*, and *PAL2*. Apart from SA content, we found that PhOBF1 silencing and overexpression led to down-regulation and up-regulation of these genes, respectively (Fig. 7C). Interestingly, silencing of R2R3-MYB-like transcription factors, *ODO1* (Verdonk *et al.*, 2005), *EOBI* and *EOBII* (Spitzer-Rimon *et al.*, 2012), also results in reduction of a set of genes transcripts in the shikimate and phenylpropanoid pathways. ODO1, transcriptionally regulated by EOBI and EOBII (Spitzer-Rimon *et al.*, 2012), is involved in floral fragrance production of petunia, and its suppression causes a decreased level of methyl salicylate (Verdonk *et al.*, 2005). It appears likely that one of these three MYB-like genes might be a crucial target of PhOBF1 for the SA-mediated RNA silencing mechanism. However, we found that the petunia plants inoculated with TRV-*PhCHS*/*ODO1* in the VIGS assay showed the *CHS*-silenced white-corollas phenotype (data not shown), suggesting that PhOBF1 is unlikely to regulate the SA-mediated antiviral RNA silencing system by activating downstream *ODO1* expression. The involvement of PhOBF1 in floral scent biosynthesis requires further examination in the future.

It should be mentioned that TRV 16 kDa (16K) protein, encoded by genomic RNA1, functions as a suppressor of RNA silencing. However, unlike the strong silencing suppressors CMV 2b (Goto *et al.*, 2007) and Tombusvirus P19 (Lakatos *et al.*, 2004), TRV 16K acts as a weak suppressor that transiently blocks local RNA silencing and enhances viral RNA accumulation early (Martínez-Priego *et al.*, 2008). In this study, we mainly focused on the silencing phenotypes in systemic leaves or flowers infected with various TRV constructs. It seems likely that the silencing of endogenous genes and antiviral defenses in inoculated leaves are more complex. In the early infection process, 16K suppressor may impose effects on PhOBF1-involved antiviral RNA silencing, and it remains unclear whether these effects can lead to the changes of final responses in systemically infected tissues. Furthermore, a possible relationship between PhOBF1 and TRV 16K suppressor should be explored in future work.

We are interested in the role of PhOBF1 in the regulation of flower senescence. Ethylene treatment increased the transcripts of *PhOBF1* (Fig. 1D). Its transcript levels were also up-regulated at various developmental stages of petunia corollas (Supplementary Fig. S5). Our analysis of flower longevity revealed that silencing of *PhOBF1* accelerated flower senescence and its overexpression extended flower longevity (Supplementary Table S3). Furthermore, both ABA and GA_3_ treatments increased the expression levels of *PhOBF1* (Fig. 1D). The crosstalk between ethylene and ABA (Chang *et al.*, 2014) or GA_3_ (Yin *et al.*, 2015) during flower senescence has been discussed in petunia. More studies are required to clarify the role of PhOBF1 in the regulation of petunia flower senescence. Additionally, the role of PhOBF1 in plant growth and development should be further investigated, because we found that down-regulation of *PhOBF1* resulted in increased plant height and reduced stem diameter, while overexpression of *PhOBF1* caused slightly smaller and thicker leaves (data not shown).

It is worth mentioning that PhERF2, an ERF transcription factor we previously reported, plays a quite similar role in antiviral RNA silencing to PhOBF1. *PhERF2* silencing also substantially impaired the *PDS*-silenced leaf photobleaching phenotype and *PhERF2* overexpression restored it (Sun *et al.*, 2016). The same involvements of PhOBF1 and PhERF2 in resistance to TRV were found. Thus, we hypothesize that a close association between PhOBF1 and PhERF2 probably occurs. In Arabidopsis, OBF4 interacts with an ethylene-responsive element binding factor AtEBP and may collaboratively contribute to the plant defense response (Büttner and Singh, 1997). However, we did not find altered *PhERF2* expression in *PhOBF1*-silenced and -overexpressing lines, or altered *PhOBF1* expression in *PhERF2* transgenic lines (data not shown). *PhOBF1* and *PhERF2* affected transcript levels of different RNA silencing-related genes, and for example, *PhOBF1* rather than *PhERF2* affected expression levels of *RDR1*, *DCL1*, and *DCL4*. Moreover, PhOBF1, not PhERF2, plays a role in the regulation of petunia flower senescence. One explanation is that they perhaps regulate TRV-induced RNA silencing through two distinct pathways. ERF proteins specifically bind to the GCC box, which commonly presents in the promoter of downstream defensive genes of the ethylene signaling pathway (Ohme-Takagi and Shinshi, 1995). It seems likely that PhOBF1 and PhERF2 may be a positive regulator in the SA biosynthesis pathway and ethylene signaling pathway, respectively.

Our data demonstrate that PhOBF1 plays an important role in antiviral RNA silencing. The positive regulation of PhOBF1 in the TRV-induced gene silencing process via SA may provide a feasible approach to improve the efficiency of the TRV-based VIGS system, through a transient overexpression of *PhOBF1* or its homologs, or an appropriate application of exogenous SA. Further confirmation should be sought for whether PhOBF1 also plays a regulatory role in other virus-based VIGS systems besides TRV. It is noteworthy that abiotic stresses, such as salt, drought, oxidation and low temperature, induce the expression of a number of RNA silencing-related genes in tomato (Bai *et al.*, 2012). We also found that low temperature and dehydration treatments increased the transcript levels of *PhOBF1* (Fig. 1C). The existing evidence supports that low temperature and low humidity enhance TRV-induced gene silencing efficiency in tomato (Fu *et al.*, 2006). The effects of these abiotic stresses on the RNA silencing response still require more study in the future.

## Supplementary data

Supplementary data are available at *JXB* online.

Fig. S1. Petunia *PhOBF1* cDNA and deduced amino acid sequence analysis.

Fig. S2. Expression of *PhOBF1* in various tissues of petunia plant.

Fig. S3. Failed development of leaf photobleaching and white-corollas phenotypes in petunia plants inoculated with a *PhPDS*/*CHS*/*OBF1* tandem TRV construct.

Fig. S4. Induction of RNA silencing-related genes in petunia leaves treated with salicylic acid.

Fig. S5. Induction of *PhOBF1* expression during petunia flower senescence.

Table S1. Primers used for semi-quantitative RT-PCR and quantitative real-time PCR.

Table S2. The numerical data for relative accumulation or expression levels of TRV RNAs and TMV-*CP* in WT, *PhOBF1*-RNAi and *PhOBF1*-overexpressing lines infected with TRV empty vector and TMV, respectively.

Table S3. The longevity of attached flowers from WT, *PhOBF1*-RNAi and *PhOBF1*-overexpressing lines.

## Supplementary Material

supplementary_figures_S1_S5_Tables_S1_S3

## References

[CIT0001] Alamillo JM Saénz P García JA 2006 Salicylic acid-mediated and RNA-silencing defense mechanisms cooperate in the restriction of systemic spread of plum pox virus in tobacco. The Plant Journal 48, 217–227. 17018032 10.1111/j.1365-313X.2006.02861.x

[CIT0002] Alvarado VY Scholthof HB 2011 AGO2: A new argonaute compromising plant virus accumulation. Frontiers in Plant Science 2, 112. 22639628 10.3389/fpls.2011.00112 PMC3355599

[CIT0003] Andika IB Maruyama K Sun L Kondo H Tamada T Suzuki N 2015 Differential contributions of plant Dicer-like proteins to antiviral defences against *Potato virus* X in leaves and roots. The Plant Journal 81, 781–793. 25619543 10.1111/tpj.12770

[CIT0004] Axtell MJ 2013 Classification and comparison of small RNAs from plants. Annual Review of Plant Biology 64, 137–159. 10.1146/annurev-arplant-050312-120043 23330790

[CIT0005] Bai M Yang GS Chen WT Mao ZC Kang HX Chen GH Yang YH Xie BY 2012 Genome-wide identification of Dicer-like, Argonaute and RNA-dependent RNA polymerase gene families and their expression analyses in response to viral infection and abiotic stresses in *Solanum lycopersicum*. Gene 501, 52–62. 22406496 10.1016/j.gene.2012.02.009

[CIT0006] Bartel B Bartel DP 2003 MicroRNAs: at the root of plant development?Plant Physiology 132, 709–717. 12805599 10.1104/pp.103.023630 PMC523861

[CIT0007] Baumberger N Baulcombe D 2005 *Arabidopsis* Argonaute1 is an RNA Slicer that selectively recruits microRNAs and short interfering RNAs. Proceedings of the National Academy of Sciences, USA 102, 11928–11933. 10.1073/pnas.0505461102 PMC1182554 16081530

[CIT0008] Bellés JM Garro R Fayos J Navarro P Primo J Conejero V 1999 Gentisic acid as a pathogen-inducible signal, additional to salicylic acid for activation of plant defenses in tomato. Molecular Plant-Microbe Interactions 12, 227–235.

[CIT0009] Bouché N Lauressergues D Gasciolli V Vaucheret H 2006 An antagonistic function for *Arabidopsis* DCL2 in development and a new function for DCL4 in generating viral siRNAs. The EMBO Journal 25, 3347–3356. 16810317 10.1038/sj.emboj.7601217 PMC1523179

[CIT0010] Burch-Smith TM Schiff M Liu Y Dinesh-Kumar SP 2006 Efficient virus-induced gene silencing in *Arabidopsis*. Plant Physiology 142, 21–27. 16815951 10.1104/pp.106.084624 PMC1557620

[CIT0011] Büttner M Singh KB 1997 *Arabidopsis thaliana* ethylene-responsive element binding protein (AtEBP), an ethylene-inducible, GCC box DNA-binding protein interacts with an ocs element binding protein. Proceedings of the National Academy of Sciences, USA 94, 5961–5966. 10.1073/pnas.94.11.5961 PMC20889 9159183

[CIT0012] Campos L Granell P Tárraga S López-Gresa P Conejero V Bellés JM Rodrigo I Lisón P 2014 Salicylic acid and gentisic acid induce RNA silencing-related genes and plant resistance to RNA pathogens. Plant Physiology and Biochemistry 77, 35–43. 24531234 10.1016/j.plaphy.2014.01.016

[CIT0013] Chang X Donnelly L Sun D Rao J Reid MS Jiang CZ 2014 A Petunia homeodomain-leucine zipper protein, PhHD-Zip, plays an important role in flower senescence. PLoS One 9, e88320. 24551088 10.1371/journal.pone.0088320 PMC3925126

[CIT0014] Chen JC Jiang CZ Gookin TE Hunter DA Clark DG Reid MS 2004 Chalcone synthase as a reporter in virus-induced gene silencing studies of flower senescence. Plant Molecular Biology 55, 521–530. 15604697 10.1007/s11103-004-0590-7

[CIT0015] Chen W Singh KB 1999 The auxin, hydrogen peroxide and salicylic acid induced expression of the *Arabidopsis* GST6 promoter is mediated in part by an ocs element. The Plant Journal 19, 667–677. 10571852 10.1046/j.1365-313x.1999.00560.x

[CIT0016] Chivasa S Murphy AM Naylor M Carr JP 1997 Salicylic acid interferes with tobacco mosaic virus replication via a novel salicylhydroxamic acid-sensitive mechanism. The Plant Cell 9, 547–557. 12237364 10.1105/tpc.9.4.547 PMC156938

[CIT0017] Costa AT Bravo JP Makiyama RK Nunes AV Maia IG 2013 Viral counter defense X antiviral immunity in plants: mechanisms for survival. In: Romanowski C, ed. Current issues in molecular virology – Viral genetics and biotechnological applications. Rijeka, Croatia: Intech, 251–285.

[CIT0018] Deleris A Gallego-Bartolome J Bao J Kasschau KD Carrington JC Voinnet O 2006 Hierarchical action and inhibition of plant Dicer-like proteins in antiviral defense. Science 313, 68–71. 16741077 10.1126/science.1128214

[CIT0019] D’Maris Amick Dempsey AC Vlot MCW Daniel FK 2011 Salicylic acid biosynthesis and metabolism. The Arabidopsis Book 9, e0156. 22303280 10.1199/tab.0156 PMC3268552

[CIT0020] Donaire L Barajas D Martínez-García B Martínez-Priego L Pagán I Llave C 2008 Structural and genetic requirements for the biogenesis of tobacco rattle virus-derived small interfering RNAs. Journal of Virology 82, 5167–5177. 18353962 10.1128/JVI.00272-08 PMC2395200

[CIT0021] Ellis JG Tokuhisa JG Llewellyn DJ Bouchez D Singh K Dennis ES Peacock WJ 1993 Does the ocs-element occur as a functional component of the promoters of plant genes?The Plant Journal 4, 433–443. 8220489 10.1046/j.1365-313x.1993.04030433.x

[CIT0022] Estrada-Melo AC Ma C Reid MS Jiang CZ 2015 Overexpression of an ABA biosynthesis gene using a stress-inducible promoter enhances drought resistance in petunia. Horticulture Research 2, 15013. 26504568 10.1038/hortres.2015.13 PMC4595983

[CIT0023] Falcioni T Ferrio JP Del Cueto AI Giné J Achón MÁ Medina V 2014 Effect of salicylic acid treatment on tomato plant physiology and tolerance to *Potato virus* X infection. European Journal of Plant Pathology 138, 331–345.

[CIT0024] Fu DQ Zhu BZ Zhu HL Zhang HX Xie YH Jiang WB Zhao XD Luo KB 2006 Enhancement of virus-induced gene silencing in tomato by low temperature and low humidity. Molecules and Cells 21, 153–160. 16511359

[CIT0025] Garcia-Ruiz H Takeda A Chapman EJ Sullivan CM Fahlgren N Brempelis KJ Carrington JC 2010 *Arabidopsis* RNA-dependent RNA polymerases and dicer-like proteins in antiviral defense and small interfering RNA biogenesis during *Turnip Mosaic Virus* infection. The Plant Cell 22, 481–496. 20190077 10.1105/tpc.109.073056 PMC2845422

[CIT0026] Goto K Kobori T Kosaka Y Natsuaki T Masuta C 2007 Characterization of silencing suppressor 2b of cucumber mosaic virus based on examination of its small RNA-binding abilities. Plant & Cell Physiology 48, 1050–1060. 17567638 10.1093/pcp/pcm074

[CIT0027] Gould B Kramer EM 2007 Virus-induced gene silencing as a tool for functional analyses in the emerging model plant Aquilegia (columbine, Ranunculaceae). Plant Methods 3, 6. 17430595 10.1186/1746-4811-3-6 PMC1855323

[CIT0028] Hanson J Hanssen M Wiese A Hendriks MM Smeekens S 2008 The sucrose regulated transcription factor bZIP11 affects amino acid metabolism by regulating the expression of *ASPARAGINE SYNTHETASE1* and *PROLINE DEHYDROGENASE2*. The Plant Journal 53, 935–949. 18088315 10.1111/j.1365-313X.2007.03385.x

[CIT0029] Harvey JJ Lewsey MG Patel K Westwood J Heimstädt S Carr JP Baulcombe DC 2011 An antiviral defense role of AGO2 in plants. PLoS One 6, e14639. 21305057 10.1371/journal.pone.0014639 PMC3031535

[CIT0030] Hull R 2009 Mechanical inoculation of plant viruses. Current Protocols in Microbiology 16B.6, doi:10.1002/9780471729259.mc16b06s13. 10.1002/9780471729259.mc16b06s13 19412912

[CIT0031] Jakoby M Weisshaar B Dröge-Laser W Vicente-Carbajosa J Tiedemann J Kroj T Parcy F; bZIP Research Group 2002 bZIP transcription factors in *Arabidopsis*. Trends in Plant Science 7, 106–111. 11906833 10.1016/s1360-1385(01)02223-3

[CIT0032] Janzik I Preiskowski S Kneifel H 2005 Ozone has dramatic effects on the regulation of the prechorismate pathway in tobacco (*Nicotiana tabacum* L. cv. Bel W3). Planta 223, 20–27. 16078071 10.1007/s00425-005-0060-8

[CIT0033] Jaubert M Bhattacharjee S Mello AF Perry KL Moffett P 2011 ARGONAUTE2 mediates RNA-silencing antiviral defenses against *Potato virus* X in *Arabidopsis*. Plant Physiology 156, 1556–1564. 21576511 10.1104/pp.111.178012 PMC3135937

[CIT0034] Ji LH Ding SW 2001 The suppressor of transgene RNA silencing encoded by Cucumber mosaic virus interferes with salicylic acid-mediated virus resistance. Molecular Plant-Microbe Interactions 14, 715–724. 11386367 10.1094/MPMI.2001.14.6.715

[CIT0035] Jiang CZ Chen JC Reid MS 2011 Virus-induced gene silencing in ornamental plants. In: Kodama H Komamine A, eds. RNAi and plant gene function analysis. New York: Humana Press, 81–96. 10.1007/978-1-61779-123-9_6 21533687

[CIT0036] Kasai M Matsumura H Yoshida K Terauchi R Taneda A Kanazawa A 2013 Deep sequencing uncovers commonality in small RNA profiles between transgene-induced and naturally occurring RNA silencing of chalcone synthase-A gene in petunia. BMC Genomics 14, 63. 23360437 10.1186/1471-2164-14-63 PMC3608071

[CIT0037] Katagiri F Chua NH 1992 Plant transcription factors: present knowledge and future challenges. Trends in Genetics 8, 22–27. 1369731 10.1016/0168-9525(92)90020-5

[CIT0038] Kumar D Klessig DF 2003 High-affinity salicylic acid-binding protein 2 is required for plant innate immunity and has salicylic acid-stimulated lipase activity. Proceedings of the National Academy of Sciences, USA 100, 16101–16106. 10.1073/pnas.0307162100 PMC307699 14673096

[CIT0039] Kusano T Berberich T Harada M Suzuki N Sugawara K 1995 A maize DNA-binding factor with a bZIP motif is induced by low temperature. Molecular & General Genetics 248, 507–517. 7476849 10.1007/BF02423445

[CIT0040] Kusano T Sugawara K Harada M Berberich T 1998 Molecular cloning and partial characterization of a tobacco cDNA encoding a small bZIP protein. Biochimica et Biophysica Acta 1395, 171–175. 9473660 10.1016/s0167-4781(97)00161-9

[CIT0041] Lakatos L Szittya G Silhavy D Burgyán J 2004 Molecular mechanism of RNA silencing suppression mediated by p19 protein of tombusviruses. The EMBO Journal 23, 876–884. 14976549 10.1038/sj.emboj.7600096 PMC381004

[CIT0042] Lee DH Choi HW Hwang BK 2011 The pepper E3 ubiquitin ligase RING1 gene, CaRING1, is required for cell death and the salicylic acid-dependent defense response. Plant Physiology 156, 2011–2025. 21628629 10.1104/pp.111.177568 PMC3149946

[CIT0043] Lewsey MG Carr JP 2009 Effects of DICER-like proteins 2, 3 and 4 on cucumber mosaic virus and tobacco mosaic virus infections in salicylic acid-treated plants. The Journal of General Virology 90, 3010–3014. 19710258 10.1099/vir.0.014555-0

[CIT0044] Liang YC Reid MS Jiang CZ 2014 Controlling plant architecture by manipulation of gibberellic acid signalling in petunia. Horticulture Research 1, 14061. 26504556 10.1038/hortres.2014.61 PMC4596332

[CIT0045] Ma J Hanssen M Lundgren K 2011 The sucrose-regulated *Arabidopsis* transcription factor bZIP11 reprograms metabolism and regulates trehalose metabolism. The New Phytologist 191, 733–745. 21534971 10.1111/j.1469-8137.2011.03735.x

[CIT0046] Ma X Nicole MC Meteignier LV Hong N Wang G Moffett P 2015 Different roles for RNA silencing and RNA processing components in virus recovery and virus-induced gene silencing in plants. Journal of Experimental Botany 66, 919–932. 25385769 10.1093/jxb/eru447

[CIT0047] Malamy J Carr JP Klessig DF Raskin I 1990 Salicylic acid: a likely endogenous signal in the resistance response of tobacco to viral infection. Science 250, 1002–1004. 17746925 10.1126/science.250.4983.1002

[CIT0048] Martínez-Priego L Donaire L Barajas D Llave C 2008 Silencing suppressor activity of the *Tobacco rattle* virus-encoded 16-kDa protein and interference with endogenous small RNA-guided regulatory pathways. Virology 376, 346–356. 18456303 10.1016/j.virol.2008.03.024

[CIT0049] Molnar A Melnyk CW Bassett A Hardcastle TJ Dunn R Baulcombe DC 2010 Small silencing RNAs in plants are mobile and direct epigenetic modification in recipient cells. Science 328, 872–875. 20413459 10.1126/science.1187959

[CIT0050] Murphy AM Carr JP 2002 Salicylic acid has cell-specific effects on tobacco mosaic virus replication and cell-to-cell movement. Plant Physiology 128, 552–563. 11842159 10.1104/pp.010688 PMC148918

[CIT0051] Nijhawan A Jain M Tyagi AK Khurana JP 2008 Genomic survey and gene expression analysis of the basic leucine zipper transcription factor family in rice. Plant Physiology 146, 333–350. 18065552 10.1104/pp.107.112821 PMC2245831

[CIT0052] Ohme-Takagi M Shinshi H 1995 Ethylene-inducible DNA binding proteins that interact with an ethylene-responsive element. The Plant Cell 7, 173–182. 7756828 10.1105/tpc.7.2.173 PMC160773

[CIT0053] Qu F Ye X Morris TJ 2008 *Arabidopsis* DRB4, AGO1, AGO7, and RDR6 participate in a DCL4-initiated antiviral RNA silencing pathway negatively regulated by DCL1. Proceedings of the National Academy of Sciences, USA 105, 14732–14737. 10.1073/pnas.0805760105 PMC2567185 18799732

[CIT0054] Quilis J Peñas G Messeguer J Brugidou C San Segundo B 2008 The *Arabidopsis* AtNPR1 inversely modulates defense responses against fungal, bacterial, or viral pathogens while conferring hypersensitivity to abiotic stresses in transgenic rice. Molecular Plant-Microbe Interactions 21, 1215–1231. 18700826 10.1094/MPMI-21-9-1215

[CIT0055] Reid MS Chen JC Jiang CZ 2009 Virus-induced gene silencing for functional characterization of genes in petunia. In: Gerats T Strommer J, eds. Petunia. Berlin Heidelberg: Springer, 381–394.

[CIT0056] Riechmann JL Heard J Martin G 2000 *Arabidopsis* transcription factors: genome-wide comparative analysis among eukaryotes. Science 290, 2105–2110. 11118137 10.1126/science.290.5499.2105

[CIT0057] Rivas-San Vicente M Plasencia J 2011 Salicylic acid beyond defence: its role in plant growth and development. Journal of Experimental Botany 62, 3321–3338. 21357767 10.1093/jxb/err031

[CIT0058] Scholthof HB Alvarado VY Vega-Arreguin JC Ciomperlik J Odokonyero D Brosseau C Jaubert M Zamora A Moffett P 2011 Identification of an ARGONAUTE for antiviral RNA silencing in *Nicotiana benthamiana*. Plant Physiology 156, 1548–1555. 21606315 10.1104/pp.111.178764 PMC3135948

[CIT0059] Schwach F Vaistij FE Jones L Baulcombe DC 2005 An RNA-dependent RNA polymerase prevents meristem invasion by *Potato virus* X and is required for the activity but not the production of a systemic silencing signal. Plant Physiology 138, 1842–1852. 16040651 10.1104/pp.105.063537 PMC1183376

[CIT0060] Shang J Xi DH Xu F 2011 A broad-spectrum, efficient and nontransgenic approach to control plant viruses by application of salicylic acid and jasmonic acid. Planta 233, 299–308. 21046144 10.1007/s00425-010-1308-5

[CIT0061] Shimizu H Sato K Berberich T Miyazaki A Ozaki R Imai R Kusano T 2005 LIP19, a basic region leucine zipper protein, is a Fos-like molecular switch in the cold signaling of rice plants. Plant & Cell Physiology 46, 1623–1634. 16051676 10.1093/pcp/pci178

[CIT0062] Spitzer-Rimon B Farhi M Albo B 2012 The R2R3-MYB-like regulatory factor EOBI, acting downstream of EOBII, regulates scent production by activating ODO1 and structural scent-related genes in petunia. The Plant Cell 24, 5089–5105. 23275577 10.1105/tpc.112.105247 PMC3556977

[CIT0063] Sun D Nandety RS Zhang Y Reid MS Niu L Jiang CZ 2016 A petunia ethylene-responsive element binding factor, PhERF2, plays an important role in antiviral RNA silencing. Journal of Experimental Botany 67, 3353–3365. 27099376 10.1093/jxb/erw155 PMC4892726

[CIT0064] Thalor SK Berberich T Lee SS Yang SH Zhu X Imai R Takahashi Y Kusano T 2012 Deregulation of sucrose-controlled translation of a bZIP-type transcription factor results in sucrose accumulation in leaves. PLoS One 7, e33111. 22457737 10.1371/journal.pone.0033111 PMC3310857

[CIT0065] Tian J Pei H Zhang S Chen J Chen W Yang R Meng Y You J Gao J Ma N 2014 TRV-GFP: a modified Tobacco rattle virus vector for efficient and visualizable analysis of gene function. Journal of Experimental Botany 65, 311–322. 24218330 10.1093/jxb/ert381 PMC3883300

[CIT0066] Tzin V Galili G 2010 The Biosynthetic pathways for shikimate and aromatic amino acids in *Arabidopsis* thaliana. The Arabidopsis Book 8, e0132. 22303258 10.1199/tab.0132 PMC3244902

[CIT0067] Van Huijsduijnen RH Alblas S De Rijk R Bol J 1986 Induction by salicylic acid of pathogenesis-related proteins and resistance to *Alfalfa mosaic virus* infection in various plant species. Journal of General Virology 67, 2135–2143.

[CIT0068] Verdonk JC Haring MA van Tunen AJ Schuurink RC 2005 ODORANT1 regulates fragrance biosynthesis in petunia flowers. The Plant Cell 17, 1612–1624. 15805488 10.1105/tpc.104.028837 PMC1091778

[CIT0069] Voinnet O 2009 Origin, biogenesis, and activity of plant microRNAs. Cell 136, 669–687. 19239888 10.1016/j.cell.2009.01.046

[CIT0070] Wang H Stier G Lin J Liu G Zhang Z Chang Y Reid MS Jiang CZ 2013 Transcriptome changes associated with delayed flower senescence on transgenic petunia by inducing expression of etr1-1, a mutant ethylene receptor. PLoS One 8, e65800. 23874385 10.1371/journal.pone.0065800 PMC3706537

[CIT0071] Wang JF Zhou H Chen YQ Luo QJ Qu LH 2004 Identification of 20 microRNAs from *Oryza sativa*. Nucleic Acids Research 32, 1688–1695. 15020705 10.1093/nar/gkh332 PMC390330

[CIT0072] Wang XB Jovel J Udomporn P Wang Y Wu Q Li WX Gasciolli V Vaucheret H Ding SW 2011 The 21-nucleotide, but not 22-nucleotide, viral secondary small interfering RNAs direct potent antiviral defense by two cooperative argonautes in *Arabidopsis* thaliana. The Plant Cell 23, 1625–1638. 21467580 10.1105/tpc.110.082305 PMC3101545

[CIT0073] Wang XB Wu Q Ito T Cillo F Li WX Chen X Yu JL Ding SW 2010 RNAi-mediated viral immunity requires amplification of virus-derived siRNAs in *Arabidopsis thaliana*. Proceedings of the National Academy of Sciences, USA 107, 484–489. 10.1073/pnas.0904086107 PMC2806737 19966292

[CIT0074] Weiste C Dröge-Laser W 2014 The *Arabidopsis* transcription factor bZIP11 activates auxin-mediated transcription by recruiting the histone acetylation machinery. Nature Communications 5, 3883. 10.1038/ncomms4883 24861440

[CIT0075] Wiese A Elzinga N Wobbes B Smeekens S 2004 A conserved upstream open reading frame mediates sucrose-induced repression of translation. The Plant Cell 16, 1717–1729. 15208401 10.1105/tpc.019349 PMC514156

[CIT0076] Wu J Yang Z Wang Y Zheng L Ye R Ji Y Zhao S Ji S Liu R Xu L 2015 Viral-inducible Argonaute18 confers broad-spectrum virus resistance in rice by sequestering a host microRNA. eLife 4, e05733. 10.7554/eLife.05733 PMC4358150 25688565

[CIT0077] Xie Z Fan B Chen C Chen Z 2001 An important role of an inducible RNA-dependent RNA polymerase in plant antiviral defense. Proceedings of the National Academy of Sciences, USA 98, 6516–6521. 10.1073/pnas.111440998 PMC33500 11353867

[CIT0078] Yang SJ Carter SA Cole AB Cheng NH Nelson RS 2004 A natural variant of a host RNA-dependent RNA polymerase is associated with increased susceptibility to viruses by *Nicotiana benthamiana*. Proceedings of the National Academy of Sciences, USA 101, 6297–6302. 10.1073/pnas.0304346101 PMC395963 15079073

[CIT0079] Yang SH Berberich T Sano H Kusano T 2001 Specific association of transcripts of tbzF and tbz17, tobacco genes encoding basic region leucine zipper-type transcriptional activators, with guard cells of senescing leaves and/or flowers. Plant Physiology 127, 23–32. 11553731 10.1104/pp.127.1.23 PMC117959

[CIT0080] Yin J Chang X Kasuga T Bui M Reid MS Jiang CZ 2015 A basic helix-loop-helix transcription factor, PhFBH4, regulates flower senescence by modulating ethylene biosynthesis pathway in petunia. Horticulture Research 2, 15059. 26715989 10.1038/hortres.2015.59 PMC4680862

[CIT0081] Zhang X Zhang X Singh J Li D Qu F 2012 Temperature-dependent survival of Turnip crinkle virus-infected arabidopsis plants relies on an RNA silencing-based defense that requires dcl2, AGO2, and HEN1. Journal of Virology 86, 6847–6854. 22496240 10.1128/JVI.00497-12 PMC3393596

