# Supplementary material for: PhOBF1, a petunia ocs element binding factor, plays an important role in antiviral RNA silencing
Source: J Exp Bot. 2017 Jan 4;68(5):915–30. doi: 10.1093/jxb/erw490 (PMC6055658; doi:10.1093/jxb/erw490)
Supplement: supplementary_figures_S1_S5_Tables_S1_S3 [file erw490_suppl_supplementary_figures_s1_s5_tables_s1_s3.pdf]

A petunia ocs element binding factor, *PhOBF1*, plays an important role in antiviral RNA silencing

Supplementary Data

A

```
                                          tatttttcttctgttactctgtctctttttcctctagctattaatttctactctttttagg
ctatttagttgctgagtttttagtcttttggctataactcattaagtatccaatctttacaccatcttattaaggactttctgaattttc
ttgtgaaaaaattcatgctgcatattggatatttattcatgtacaagattcatgtctcctgttcttagtgaaatccttcgttctggtttt
                                                            M S P V L S E I L R S G F
ataatcagttcttcccttagacgcaggacacaccttgttcaatctttatcagttgttttcctttattggttttatgttttctcagaaata
 I I S S S L R R R T H L V Q S L S V V F L Y W F Y V F S E I
atcatcacttccccttaaaataattagcttgatctagcatttttacatagtttttatcataatctcttgtttttttttaggtgctgcatg
 I I T S P
ttttcttggtactaatatttattcaacaagaaagtcaagccgtgcctaaaattttaaaaagtgtacatatagaatattattattgttact
atggcatcttctagtggaaattcatcaggttcaatgggttcagaagaagatatgcaaggattaatgatggtggatgaaaggaaaagaaag
 M A S S S G N S S G S M G S E E D M Q G L M M V D E R K R K
agaatgcaatcaaatcgtgaatcagcaagaagatcaagaatgcgaaagcaaaaacatcttgatgatttaattggacaagtgtcacaacta
 R M Q S N R E S A R R S R M R K Q K H L D D L I G Q V S Q L
aagaaggaaaatggacacatacttagtaccatcaacatgactacaacacaatatgctaatgttgaagctgaaaattctgttttaagagca
 K K E N G H I L S T I N M T T T Q Y A N V E A E N S V L R A
caaatgatggaattaagtcaaaggttgcaatctcttaatgacatcctcaattacatcaattccaacaacaacaacaacaacaacaacaac
 Q M M E L S Q R L Q S L N D I L N Y I N S N N N N N N N N N
gttgttaatactactaatggaatatttgatttgaaactactgctcatcagcaacaagaggaaccatggaacttgatgtatttaaatcag
 V V N T T N G I F D F E T T A H Q Q Q E E P W N L M Y L N Q
ccaattatggcttcagctgatatgttctatcagtattgattattttaaaaaatttgacactatcacta
 P I M A S A D M F Y Q Y
```

B

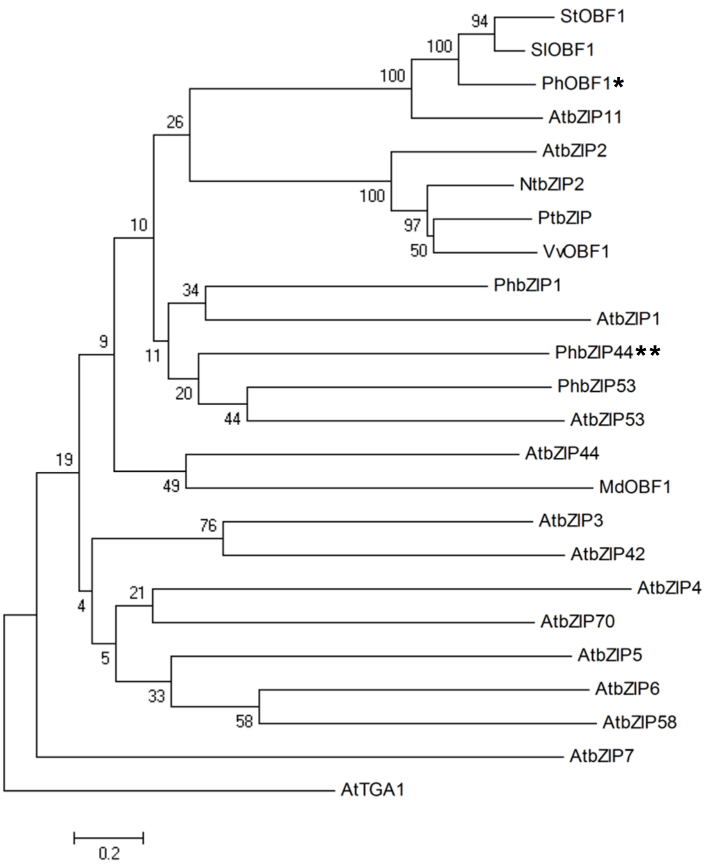

Fig. S1. Petunia *PhOBF1* cDNA and deduced amino acid sequence analysis. (A) 1029-bp cDNA sequence of *PhOBF1* contains a 489-bp open reading frame (ORF) region, encoding a protein of 162 amino acids. Putative sucrose-controlled upstream open reading frame (uORF) region is shaded in light grey. The conserved bZIP domain from amino acids 25 to 87 is shaded in dark grey. Bold italic types in square indicate the start and stop codons of translation. Solid underline marks the 330-bp fragment used for VIGS and RNAi construct, and dashed underline denotes the 268-bp fragment (fragment 2) used for VIGS confirmation. (B) Phylogenetic analysis of PhOBF1 aligned with similar proteins from Arabidopsis (group S bZIP proteins), petunia, and other plant species. The proteins analyzed include Arabidopsis AtbZIP1 (At5g49450), AtbZIP2 (At2g18160), AtbZIP3 (At5g15830), AtbZIP4 (At1g59530), AtbZIP5 (At3g49760), AtbZIP6 (At2g22850), AtbZIP7 (At4g37730), AtbZIP11 (At4g34590), AtbZIP42 (At3g30530), AtbZIP44 (At1g75390), AtbZIP53 (At3g62420), AtbZIP58 (At1g13600), AtbZIP70 (At5g60830), *Solanum tuberosum* StOBF1 (XP006341974), *Solanum lycopersicum* SlOBF1 (XP004238299), *Nicotiana tomentosiformis* NtbZIP2 (XP009618859), *Populus trichocarpa* PtbZIP (XP002306888), *Vitis vinifera* VvOBF1 (XP003634372), and *Malus domestica* MdOBF1 (XP008377201), as well as three petunia paralogs of PhOBF1, designated PhbZIP1 (FN003637), PhbZIP44 (FN023747), and PhbZIP53 (FN021185). PhOBF1 and PhbZIP44 (VIGS control) are highlighted by single and double asterisks respectively. AtTGA1 (At5g65210), One member of Arabidopsis group D bZIPs, served as the out-group. Boot-strap values were calculated based on 1000 replicates and indicated at corresponding branch nodes.

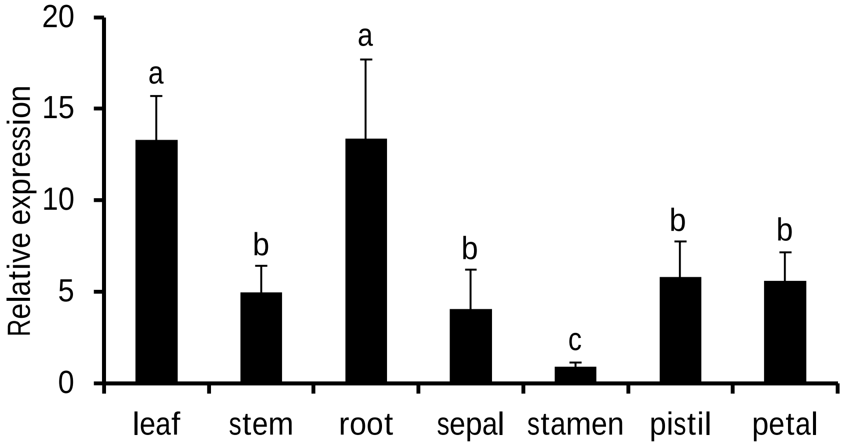

Fig. S2. Expression of *PhOBF1* in various tissues of petunia plant. Quantitative real-time PCR analysis of *PhOBF1* transcript levels in younger leaves, stems, roots, and flowers at anthesis (sepals, stamens, pistils, and petals) of 10-week-old petunia plants. Expression levels were normalized to *26S rRNA*. Error bars indicate SE of the means from three biological replicates. Different letters denote statistical significance using Duncan's multiple range test at $P<0.05$.

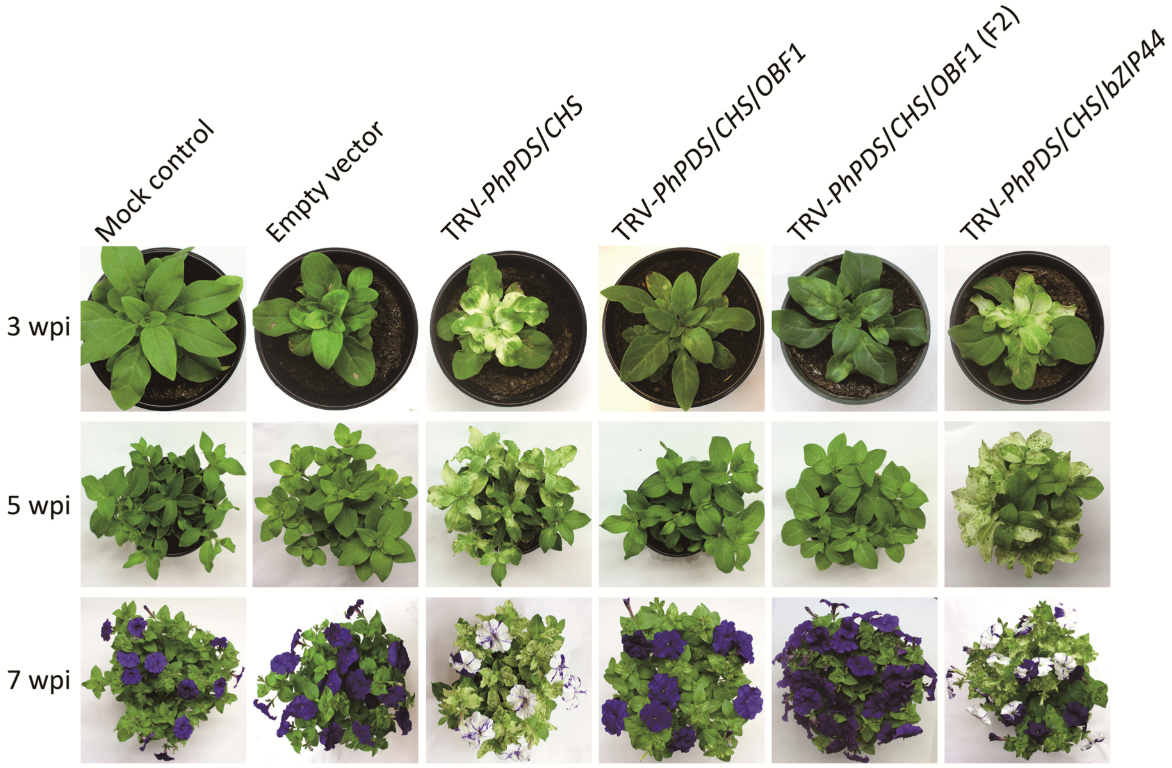

Fig. S3. Failed development of leaf photobleaching and white-corollas phenotypes in petunia plants inoculated with a *PhPDS*/*CHS*/*OBF1* tandem TRV construct. Representative phenotypes of WT plants at 3, 5, and 7 weeks post-inoculation (wpi) inoculated with non-transformed *Agrobacterium* (mock control), or *Agrobacterium* bearing a TRV empty vector, TRV-*PhPDS*/*CHS*, TRV-*PhPDS*/*CHS*/*OBF1*, TRV-*PhPDS*/*CHS*/*OBF1* (fragment 2, F2), and TRV-*PhPDS*/*CHS*/*bZIP44*.

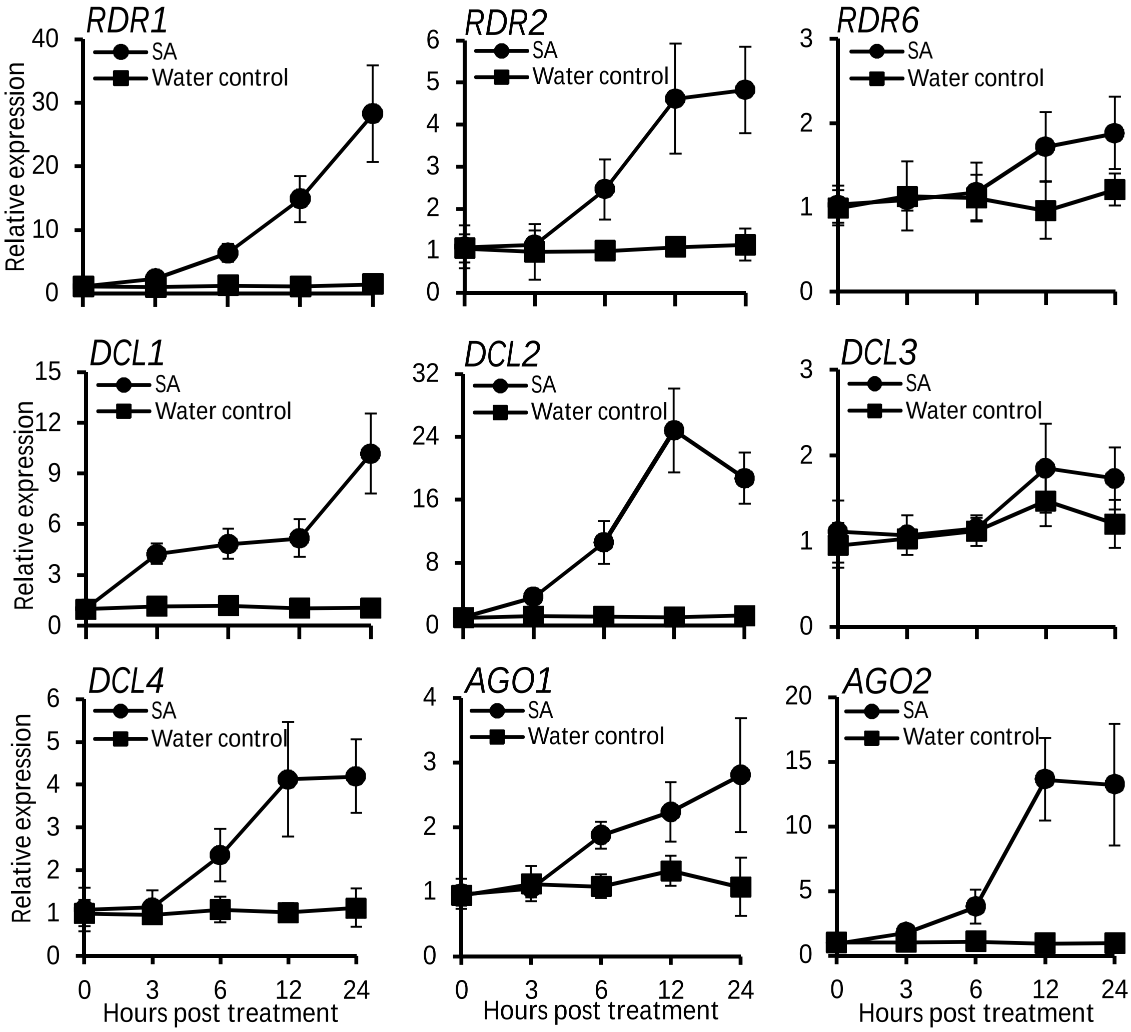

Fig. S4. Induction of RNA silencing-related genes in petunia leaves treated with salicylic acid. Quantitative real-time PCR analysis of RNA silencing-related genes, including *RDR1*, *RDR2*, *RDR6*, *DCL1*, *DCL2*, *DCL3*, *DCL4*, *AGO1*, and *AGO2*, transcript levels in the leaves treated with 200 μM SA at various time points. The treatment with distilled water was used as control. Transcript abundances were normalized to *26S rRNA*. Error bars represent SE of means from three biological replicates.

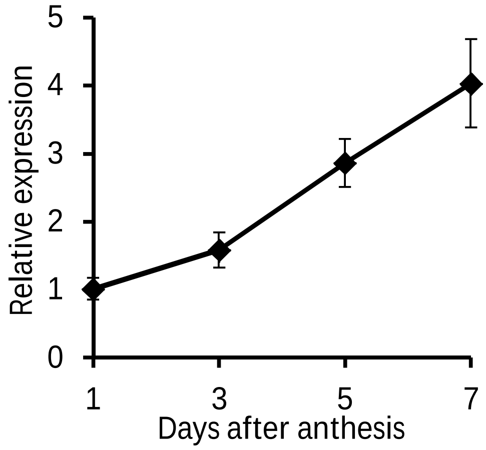

Fig. S5. Induction of *PhOBF1* expression during petunia flower senescence. Quantitative real-time PCR analysis of *PhOBF1* expression levels in the corollas at day 1, 3, 5, and 7 after anthesis. Relative expression was standardized to *26S rRNA*. Error bars represent SE of the means from three biological replicates.

Table S1. Primers used for semi-quantitative RT-PCR and quantitative real-time PCR.

Gene ID	Accession no.	Forward primer (5'-3')	Reverse primer (5'-3')	Product size
PhOBF1(F1, R1)	FN001301	AGTGAAATCCTTCGTTCTGGTTT	CATTGAACCTGATGAATTTCCAC	330bp
PhOBF1(F2, R2)	FN001301	AACATGACTACAACACAATATGC	GATAGAACATATCAGCTGAAGCC	268bp
PDS(F1, R1)	AY593974	CAGTGCTTCTTGATCGCTTTGAA	TCTGACTTGGCCACCTTTTGACT	138bp
PDS(F2, R2)	AY593974	CGAATAAGAAAGATCGAGCTGAA	CAGGCAAAAGAAGCTTGAAAATA	130bp
CHS	X14599	ACCATTGGGCATTTCTG	AGCCTTTCTCATTTCATCC	194bp
PhbZIP44	FN030231	AAGCAGAAGCATTGTGATGATTT	ATAATGGACTGGTTGACATGGAG	338bp
RNA1	AF406990	CAGTCTATACACAGAAACAGA	GACGTGTGTACTCAAGGGTT	463bp
RNA2-1(F1, R1)	AF406991	GGTTACTAGCGGCACTGAATAGA	TAGTACTCCCTTGGTTCGTCGTA	225bp
RNA2-2(F2, R2)	AF406991	ACGGACGAGTGGACTTAGATTC	GTTTAATGTCTTCGGGACATGC	Depending on insert
RDR1	CV299561	TCATGATGTCACAATTGAGGAAG	TTCGGAGTGATAGGTTGTCTTGT	271bp
RDR2	FN008007	ACGATATGGTGGTTACAAAGGTG	ATCCTCCACTCCCAAAGTAGAAA	190bp
RDR6	GT007757	TTTGCTGATCGGGAACTATTAAA	CATATGACTTTCTTGCCACTTCC	277bp
DCL1	FN028672	GGAGAAAGTTGAACAGGATGATG	CAACATCGACATCCAATACAATG	412bp
DCL2	FN020290	TGGAACCTCTGACATCGTAGAAT	ATATTCTGGAGGAAGAAGCCATC	322bp
DCL3	GBDQ0103 7961	GGAGACTTGTAAATTCTACGATC	TTTCAAGGCAACACAATATCTTT	206bp
DCL4	FN002500	AGGATATGATGAAACACCCTGTG	TAGCCTTTGAACCTGCATTAGAG	222bp
AGO1	CV298592	TCTATCAAAGTATCCGCCCTACA	CTTGAGATGTTAAACCCGAGATG	246bp
AGO2	FN018838	GTTCATCGGAGCTGATGTTAATC	GAAATAGCCGTGTATGGTGTCTC	371bp
DAHPS	JQ955569	TACTATGATTGCTCTGCCCATTT	GAGGAAGCTTAACCCTCATGTTT	235bp
SK1	FN000522	GACCATTTGACACACAAGTTGAA	GTTCACCGAGTTAATCTCCCTCT	243bp
EPSPS	M21084	GTTGATGGTCTTAAACAGCTTGG	TGATTTCAATCTCCACATCTCCT	193bp
CS	CV299364	ACTTAAGGTTGGTCCACTGCATA	TGTATGCTGTTCCCAATTAATCC	269bp
CM1	FN026368	CTATGGGAAATTTGTTGCTGAAG	TTCTTGTCCGTATGTTCTGGTTT	169bp

ADT1	FJ790412	AGTCGCATTTCAAGCTGTAGAAC	AGTGCTAAGAGGCAGTGATGAAC	165bp
PAL1	AY705976	TTTGGTGCAACATCACATAGAAG	ACCAAGTCACCAGATGCTGTAAT	281bp
PAL2	CO805160	GACCATTTGACACACAAGTTGAA	GTTCACCGAGTTAATCTCCCTCT	243bp
TMV-*CP*	KJ438787	TCAGTTCGTGTTCTTGTCATCAG	TCTAGTGTCGAATGCACCTAACA	247bp
26S rRNA	AF479174	AGCTCGTTTGATTCTGATTTCCAG	GATAGGAAGAGCCGACATCGAAGG	185bp

Table S2. The numerical data for relative accumulation or expression levels of TRV RNAs and TMV-*CP* in WT, *PhOBF1*-RNAi and *PhOBF1*-overexpressing lines infected with TRV empty vector and TMV, respectively.

	Relative accumulation or expression levels						
	WT	RNAi (2)	RNAi (6)	RNAi (8)	OE (B)	OE (D)	OE (H)
TRV RNA1	1.08±0.26	9.66±1.45	11.34±3.24	8.93±1.46	0.24±0.12	0.22±0.13	0.16±0.09
TRV RNA2	1.07±0.34	2.82±0.65	3.22±0.59	3.17±0.43	0.21±0.11	0.16±0.10	0.17±0.16
TMV-*CP*	1.01±0.21	8.22±1.97	9.10±2.29	4.51±1.13	0.24±0.18	0.21±0.08	0.16±0.13

Table S3. The longevity of attached flowers from WT, *PhOBF1*-RNAi and *PhOBF1*-overexpressing lines.

	Flower longevity (days±SD)
Wild-type	7.2[a]±0.51
PhOBF1-RNAi (2)	5.3[b]±0.68
PhOBF1-RNAi (6)	5.6[b]±0.37
PhOBF1-RNAi (8)	5.8[b]±0.60
PhOBF1-OE (B)	8.4[c]±0.37
PhOBF1-OE (D)	8.4[c]±0.49
PhOBF1-OE (H)	8.6[c]±0.86

For each line, 15 corollas from each of three individual plants were used for testing flower longevity. The recorded longevity represents the time from anthesis to completed wilting of the corolla.
